# Fixation dynamics of beneficial alleles in prokaryotic polyploid chromosomes and plasmids

**DOI:** 10.1093/genetics/iyac121

**Published:** 2022-08-12

**Authors:** Mario Santer, Anne Kupczok, Tal Dagan, Hildegard Uecker

**Affiliations:** Research Group Stochastic Evolutionary Dynamics, Department of Evolutionary Theory, Max Planck Institute for Evolutionary Biology, 24306 Plön, Germany; Institute of General Microbiology, Kiel University, 24118 Kiel, Germany; Bioinformatics Group, Department of Plant Sciences, Wageningen University & Research, 6708PB Wageningen, The Netherlands; Institute of General Microbiology, Kiel University, 24118 Kiel, Germany; Research Group Stochastic Evolutionary Dynamics, Department of Evolutionary Theory, Max Planck Institute for Evolutionary Biology, 24306 Plön, Germany

**Keywords:** polyploidy, plasmid copy number, prokaryote evolution, genetic variation, heterozygosity

## Abstract

Theoretical population genetics has been mostly developed for sexually reproducing diploid and for monoploid (haploid) organisms, focusing on eukaryotes. The evolution of bacteria and archaea is often studied by models for the allele dynamics in monoploid populations. However, many prokaryotic organisms harbor multicopy replicons—chromosomes and plasmids—and theory for the allele dynamics in populations of polyploid prokaryotes remains lacking. Here, we present a population genetics model for replicons with multiple copies in the cell. Using this model, we characterize the fixation process of a dominant beneficial mutation at 2 levels: the phenotype and the genotype. Our results show that depending on the mode of replication and segregation, the fixation of the mutant phenotype may precede genotypic fixation by many generations; we term this time interval the heterozygosity window. We furthermore derive concise analytical expressions for the occurrence and length of the heterozygosity window, showing that it emerges if the copy number is high and selection strong. Within the heterozygosity window, the population is phenotypically adapted, while both alleles persist in the population. Replicon ploidy thus allows for the maintenance of genetic variation following phenotypic adaptation and consequently for reversibility in adaptation to fluctuating environmental conditions.

## Introduction

Genetic variation is an important determinant of a population’s capacity to adapt to novel environmental conditions. In monoploid organisms, genetic variation exists only at the level of the population, whereas polyploid organisms may also be genetically heterogeneous at the intracellular level. In diploid eukaryotic organisms, observed heterozygosity—the carriage of different alleles by the 2 copies of a chromosome within a cell—is an important measure of genetic variation. In contrast, the existence and importance of intracellular genetic variation in prokaryotes have been so far much less appreciated; nonetheless, polyploidy is common in prokaryotic species (Soppa 2022). Polyploid chromosomes have been described across a wide range of taxa, including cyanobacteria (Griese *et al.* 2011; Watanabe 2020), gammaproteobacteria (Ionescu *et al.* 2017), as well as halophilic and methanogenic archaea (Breuert *et al.* 2006; Hildenbrand *et al.* 2011; Soppa 2017). The number of chromosome copies in prokaryotes ranges from a few to several hundreds and may also depend on the growth phase and the nutrient conditions (e.g. Maldonado *et al.* 1994; Hildenbrand *et al.* 2011; Watanabe 2020). In bacterial species that are monoploid during slow growth, the number of chromosomes may temporarily increase during exponential growth (Nielsen *et al.* 2007; Sun *et al.* 2018). Indeed, the early studies of bacterial genetics already observed heterozygosity in seemingly monoploid bacterial species such as *Escherichia coli* (Morse *et al.* 1956), *Bacillus subtilis* (Iyer 1965), or *Streptococcus pneumoniae* (Guerrini and Fox 1968). In addition to chromosomes, extra-chromosomal genetic elements, such as bacterial plasmids, are often present in multiple copies in the cell. The plasmid copy number depends on the plasmid type and the environmental conditions, with some plasmid types reaching hundreds of plasmid copies in the cell (Friehs 2004; Rodríguez-Beltrán *et al.* 2021).

In sexually reproducing eukaryotes, heterozygosity is typically generated at the formation of zygotes. In prokaryotes, heterozygosity is generated through de novo mutations or recombination with DNA acquired through lateral transfer. The subsequent maintenance of heterozygosity over time depends on the allele dynamics in the population. Two key determinants of allele dynamics in the population are the mode of replicon inheritance and the fitness effect of the mutant allele. Depending on the mode of replicon inheritance, daughter cells may be exact replicates of the mother cell or differ in the distribution of alleles. In the latter case, the segregation of the mutant allele may lead to the emergence of homozygous mutant cells. If the mutation is beneficial and survives stochastic loss while rare, the mutant allele will then ultimately fix in the population. Processes occurring at the intracellular level during cell division thus play an important role in the evolutionary dynamics of alleles in multicopy replicons and in their fixation processes and times.

The process of beneficial allele fixation plays a role in the rate of adaptation and the maintenance of variation. During the fixation process, both novel and wild-type alleles coexist in the population; once the beneficial allele has been fixed in the population, genetic variation at the allele locus is eliminated. Modeling allele fixation times has a long history in mathematical population genetics dating back to Kimura and Ohta (1969). Most existing models, however, focus on allele fixation in diploid sexually reproducing or in monoploid species. A recent modeling study on the evolutionary dynamics of alleles in multicopy plasmids suggests that the fixation times of alleles emerging in high-copy-number plasmids are longer than those of alleles emerging in low-copy number plasmids (Ilhan *et al.* 2019). Furthermore, Halleran *et al.* (2019) point out that random segregation of plasmid copies allows for allele fixation, while deterministic segregation hinders allele fixation. Both results clearly show that the allele dynamics on multicopy replicons are strongly influenced by the replicon properties. Yet, the effect of different replication and segregation modes on the fixation process, depending on the strength of selection, is still poorly understood.

Here, we develop a mathematical framework to model the fixation process of beneficial alleles on multicopy replicons in asexual unicellular organisms. Our framework is germane to the evolutionary dynamics of alleles in polyploid prokaryotic chromosomes and multicopy plasmids. We apply a classical population genetics model—the time-continuous Moran model with selection—and include various modes of replication and segregation of multicopy replicons. With this model, we investigate the dynamics of dominant beneficial alleles in the population. In the analysis, we follow the frequencies of heterozygous and homozygous mutant cells throughout the fixation process. The fixation of the mutation in our model is defined at the level of the cell phenotype and genotype, and we compare the fixation times at both levels. The fixation of the mutant phenotype implies phenotypic adaptation of the population. We describe that—if the 2 fixation times do not coincide—genetic variation persists in the population during the time between fixation of the phenotype and fixation of the genotype.

## The Model

**Fig. 1.**
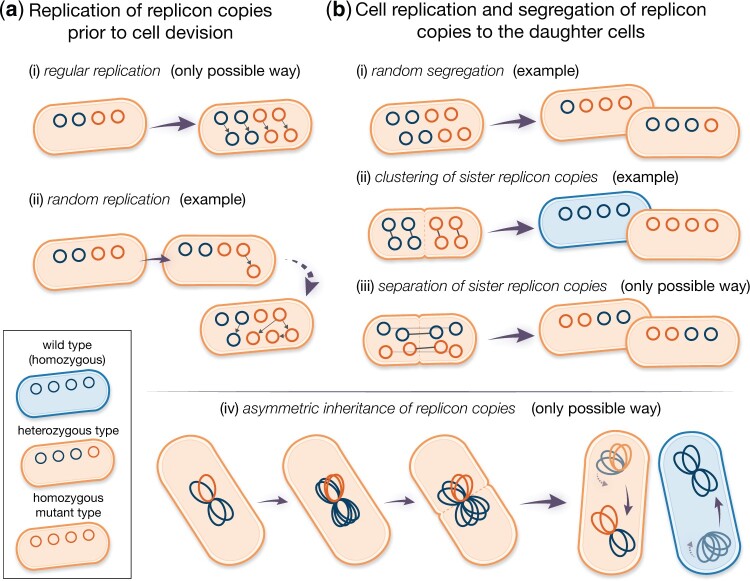
Modes of replication (a) and segregation (b) of the replicon copies modeled here. Blue and orange circles denote wild-type and mutant replicon copies, respectively. Small gray arrows between replicon copies indicate sister replicons, i.e. one replicon copy is a direct duplicate of the other. a) In case of regular replication, all replicon copies are duplicated exactly once before cell division. For random replication, replication of copies is a successive process. Random replication can lead to many different compositions of the replicon pool before cell division. b) Given the mode of random segregation, different types of daughter cells can emerge. For *clustering of sister replicon copies*, all pairs of sister replicons (gray arrows) consisting of a template and its direct duplicate are inherited to the same daughter cell. The opposite holds for *separation of sister replicon copies*, where the composition of both daughter cells is identical due to partitioning of sister copies. In case of (iv) *asymmetric inheritance of replicon copies*, clusters of replicon copies are resolved at the deepest point in their genealogy [(iv) adapted from Sun *et al.* (2018)].

We consider a population of bacteria (or other prokaryotes) with a constant number of *N* cells, each carrying *n* copies of a replicon [e.g. a multicopy (polyploid) chromosome or plasmid]. We assume that there are 2 genetic variants of the replicon, carrying the *wild-type* and the *mutant* alleles, respectively. Consequently, for *n *>* *1, cells might be either heterozygous (i.e. carrying both alleles) or homozygous (see Fig. 1).

We assume that cells carrying at least one mutant replicon copy have a selective advantage *s *>* *0. The mutation is thus dominant. Initially at *t *=* *0, the mutant type is present in a single replicon copy in a small fraction *f* of cells. The initial population composition, as defined here, may arise, for example, due to a transformation event in the laboratory (or plasmid invasion). Later, we also consider adaptation starting from a balance between recurrent transformation and purifying negative selection against mutant cells that has established in a different environment, in which the mutation is deleterious. We do not consider the emergence of de novo mutations during the fixation process.

To describe the allele dynamics, we apply a classical population genetics model, the Moran model in continuous time (Moran 1958), extended for multicopy replicons (Santer and Uecker 2020). Mutant cells with *i *>* *0 mutant replicon copies divide at rate $\lambda_{i}\equiv 1+s$, while wild-type cells divide at rate $\lambda_{0}\equiv 1$. A dividing cell (parental cell) gives rise to 2 daughter cells, which replace the parental cell and one additional, randomly chosen, cell in the population. The formation of *n* new replicon copies occurs in the model prior to cell division. Here, we consider 2 modes of replication: regular replication and random replication (Fig. 1a, see also Novick and Hoppensteadt 1978). In the regular replication mode, each replicon copy is duplicated prior to cell division. This is assumed for the replication of many chromosome types (Skarstad *et al.* 1986; Nordström and Dasgupta 2006). In the random replication mode, the following procedure is repeated *n* times: a single replicon copy is randomly selected for replication, and the replicated copy is added to the replicon pool. This mode better reflects the replication mechanism of plasmids (Rownd 1969; Bogan *et al.* 2001; Nordström 2006). Possibly but not necessarily, it might also reflect the replication of some polyploid chromosomes as in some cyanobacteria, where only a few chromosome copies are duplicated at once (Watanabe *et al.* 2012; Ohbayashi *et al.* 2019; Soppa 2022). At cell division, the total replicon pool is divided equally between the daughter cells, i.e. each daughter cell receives *n* copies. In our baseline model, we assume that the segregation of mutant and wild-type replicons to the daughter cells is random [Fig. 1b(i)]. Mathematically speaking: *n* copies are drawn from the pool of 2*n* replicon copies of the parental cell without replacement and segregate to the first daughter cell; the remaining *n* copies are segregated to the second daughter cell. Chromosome segregation is random or at least partially so in a range of bacterial and euryarchaeotic species (Hu *et al.* 2007; Schneider *et al.* 2007; Tobiason and Seifert 2010; Li 2019). This mode moreover mimics the segregation of high-copy number plasmids (Ishii *et al.* 1978; Novick and Hoppensteadt 1978; Cullum and Broda 1979). Note that randomness in segregation in our model refers to the random segregation of replicon variants. Yet, segregation of high-copy number plasmids includes in addition randomness in the number of copies that each daughter cell inherits (Münch *et al.* 2015). Likewise, active partitioning systems in low-copy number plasmids may only guarantee that no plasmid-free cells are generated but do not necessarily imply equal plasmid copy numbers in both daughter cells following cell division. We simplify this in our model to keep the number of cell types manageable.

In addition to the baseline model, we consider 3 further modes of segregation: (ii) *clustering of sister replicon copies*, (iii) *separation of sister replicon copies*, and (iv) *asymmetric inheritance of replicon copies* (Fig. 1b). Sister replicon copies are pairs where one copy is the direct replicate of the other. We only consider those in combination with regular replication, which is in some cases biologically motivated [e.g. for mode (iv)] and in others mathematical convenience [e.g. for mode (iii)].

In the segregation mode termed *clustering of sister replicon copies* (ii), sister replicons are inherited to the same daughter cell, while in the segregation mode termed *separation of sister replicon copies* (iii), the sister replicons segregate into different daughter cells. *Clustering of sister replicon copies* may happen in the presence of DNA-binding regulatory elements (Wu *et al.* 1992), which has been recently shown to affect plasmid allele segregation under nonselective conditions (Garoña *et al.* 2021). It could also serve as a rough proxy of chromosome segregation when chromosome copies are spatially sorted in the cell as in *Synechococcus elongatus* (Jain *et al.* 2012). In this mode (ii), we only consider even copy numbers *n* to be able to fulfill the assumption of equal copy numbers in both daughter cells after cell division. The separation of sister replicon copies (iii) assumes that sister replicons are well-separated postreplication, as recently shown for haploid *B. subtilis* chromosomes (Wang *et al.* 2017). The replicon separation may be achieved by active partition systems that push the replicons to the opposite cell poles such that they end up in different daughter cells at cell division, which is encoded in many low-copy-number plasmids (Nordström and Gerdes 2003; Million-Weaver and Camps 2014; Brooks and Hwang 2017). *Asymmetric inheritance of replicon copies* (iv) has been proposed by Sun *et al.* (2018) as a model for segregation of chromosomes in fast-growing bacteria, which harbor multiple chromosome copies due to multifork replication (Nielsen *et al.* 2007; Sun *et al.* 2018). Here, the replicon copy number *n* is restricted to powers of 2. In this mode, all replicon copies remain attached to each other and form one large cluster. At cell division, only the oldest link between the replicon copies is resolved so that *n* copies are inherited to every daughter cell. Effectively, this means that one of the daughter cells of a heterozygous progeny cell receives all mutant copies. A mathematical description of the model is given in Section A1.

In our model, we track the fraction of cells carrying *i* mutant replicon copies over time *t*, which we denote by $x_{i}(t)$. A time unit corresponds to the mean generation time of wild-type cells. For most of the analysis, we study the deterministic dynamics arising from large population sizes *N*, which are given by a system of *n *+* *1 ordinary differential equations [Equation (A11), Appendix A2]. We numerically integrate these equations using the Python package SciPy (Function solve_ivp, see Appendix A2 for details). We determine the proportion of heterozygous cells $x_{\text{het}}\equiv x_{1}+\cdots +x_{n-1}$ and the proportion of homozygous mutant cells *x_n_* for all times *t*.

## Results

To describe the fixation dynamics, the population-wide frequency of the mutant replicon is reported at 2 levels: the phenotype level and the genotype level. Since we consider a dominant mutant allele, all cells that carry at least one mutant replicon copy have the same phenotype (i.e. fitness in our context). The fixation at the phenotype level may not be permanently reached as long as the wild-type allele remains in the population since new wild-type homozygous cells can be regenerated at divisions of heterozygous cells. Let us ignore this for a moment and denote the time by which (nearly) all cells contain at least one mutant replicon copy by *t*_phen_. From the time point of mutant phenotype fixation, *t*_phen,_ selection is restricted to the dynamics of wild-type homozygous cells that are newly generated at cell division of heterozygous cells and their few progenitors. Otherwise, the process is neutral. The neutral dynamics and the allele segregation process, followed by purging of wild-type homozygotes, continue until no cells carrying the wild-type replicon variant are left, i.e. the population is entirely composed of homozygous mutant cells. At time *t*_fix,_ the mutation is fixed at the genotype level, and the wild-type variant has been lost from the population. In the deterministic model with a continuous state space, true fixation never occurs, and we define $t_{\text{phen}}$ as the time by which a threshold fraction $x_{\text{thr}}$ of cells contain at least one mutant replicon copy and $t_{\text{fix}}$ as the time by which a fraction $x_{\text{thr}}$ of cells are mutant homozygotes. Although $x_{\text{thr}}\neq 1$ (but only close to one), we speak of fixation if the respective population size has reached the threshold $x_{\text{thr}}$, which we set to 99% for all numerical results in the main text.

### Phenotypic and genotypic fixation times can differ for multicopy replicons, leading to a “heterozygosity window”

**Fig. 2.**
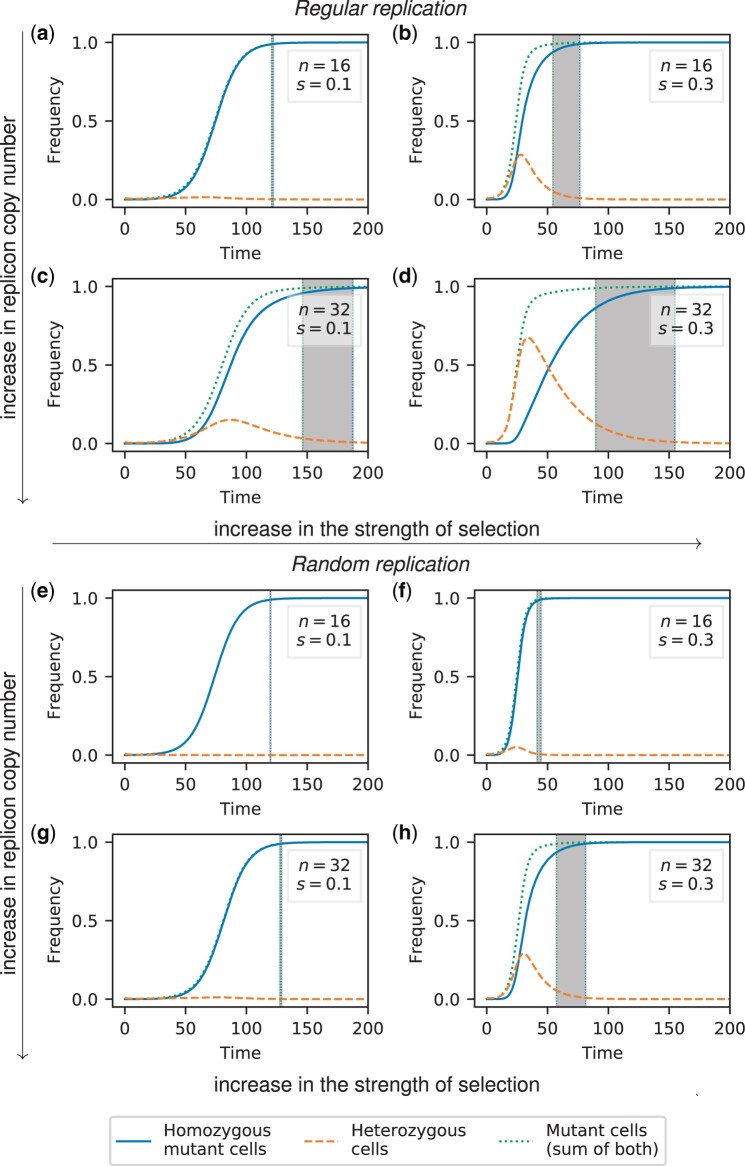
Frequency trajectories of different cell types for random segregation and regular replication (a–d) or random replication (e–h). The initial population at *t *=* *0 comprises a small fraction *f *=* *0.01 of heterozygous cells with one mutant replicon copy each. The gray area highlights the heterozygosity window, defined as the time between fixation of the mutation at the phenotype level (99% of cells carry at least one mutant copy) and fixation at the genotype level (99% of cells are mutant homozygotes). The time unit corresponds to the generation time of the wild type. Results for cell frequencies were obtained from numerical integration of the ordinary differential Equation (A11).

Notably, fixation of the mutant allele at the genotype level can occur a long time after its fixation at the phenotype level; here, we term the time interval between these 2 events the *heterozygosity window* (Fig. 2). The length $\Delta t=t_{\text{fix}}-t_{\text{phen}}$ of the heterozygosity window is important since, during this phase, the population is (almost) fully adapted; yet, genetic variation is preserved. This may enable the population to quickly adapt if the selection pressure is reversed and the wild type becomes beneficial. Of course, the potential to readily respond to this new change ultimately also depends on the dominance or recessiveness of the wild-type allele under the reversed conditions and the genotype composition of the subpopulation of cells that still carry the wild-type allele. The total time during which genetic variation persists in the population, either within cells (heterozygosity) or across cells, is given by the genotypic fixation time. (With our approximate definition of fixation in the deterministic model, some variation still exists at $t_{\text{fix}}$.) We highlight the length of the heterozygosity window because it is an important characteristic of the allele fixation dynamics in polyploid replicons. The length of the heterozygosity window tells us whether the phenotypic and genotypic fixation times are close to each other (maybe even identical) or very different. Knowing whether both times coincide or not is especially important if we only have information about fixation either on the phenotypic or the genotypic level in a population. The length of the window furthermore provides information on the ability of the population to respond to fluctuating environments.

**Fig. 3.**
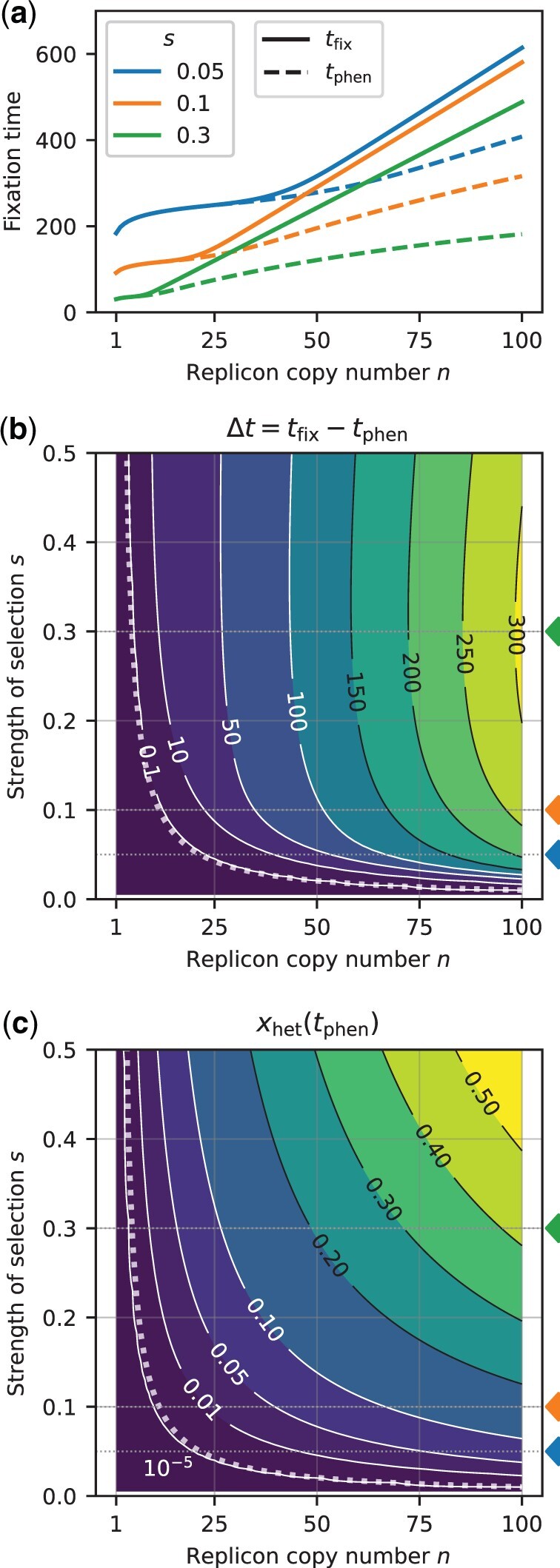
Effect of the replicon copy number *n* and the strength of selection *s* on the fixation times and the heterozygosity window for a replicon subject to regular replication and random segregation. The initial frequency of mutant cells with one mutant replicon copy at *t *=* *0 is *f *=* *0.01. a) Fixation times as a function of the replicon copy number for several selection coefficients *s *=* *0.05 (blue), 0.1 (orange), 0.3 (green) (indicated by colored triangles in b and c). Lines are for guidance of the eye; the replicon copy number *n* is discrete. b) Contour plot of the heterozygosity window for various replicon copy numbers *n* and selection coefficients *s*. The dotted line shows the threshold of *s* (as a function of *n*) at which the heterozygosity window starts to occur [criterion (1)]. c) Frequency of remaining heterozygotes at the time point of phenotypic fixation *t*_phen_. All graphs show results of deterministic numerical simulations for regular replication and random segregation [Equation (A11)]. Fixation times *t*_phen_ and *t*_fix_ were determined as the time point when mutant and homozygous mutant cells reach a threshold of 0.99, respectively.

We find that a heterozygosity window appears if the replicon copy number *n* and the strength of selection *s* are sufficiently large (Figs. 2 and 3). If *n* and *s* are large, heterozygous cells rise considerably in frequency before homozygous mutant cells become frequent and take over the population (Fig. 3c). We can use this insight to derive a condition for the existence of a heterozygosity window. We find that for regular replication and small initial frequencies (f≪1) of heterozygous cells, heterozygous cells initially increase in frequency if

(1) $$s>\frac{1}{n-\frac{3}{2}}\approx \frac{1}{n}\;\Leftrightarrow \;sn≳1,$$

where the last approximation holds for n≫1 (see Appendix A3 for a mathematical derivation). If *s* is only slightly larger than this threshold, heterozygotes barely rise before they decline, and the heterozygosity window remains very small. At the same time, even if heterozygotes decline right from the start, there is still a very small frequency of heterozygotes left at the time of phenotypic fixation, but this frequency is too low to lead to a non-negligible window. Overall, condition (1) predicts well the boundary in the *s*-*n* plane between areas with and without a non-negligible heterozygosity window (Fig. 3b, Supplementary Fig. 3a). A closer look shows that a visible window opens for slightly larger *sn* than predicted (Supplementary Fig. 2a). However, condition (1) becomes accurate in the limit $x_{\text{thr}}\to 1$ (see Supplementary Fig. 2b for an example with a larger threshold and the comment on the convergence of the window size for $x_{\text{thr}}\to 1$ at the end of this section).

If multicopy replicons undergo random rather than regular replication, the threshold of *s* and *n* for a heterozygosity window to appear is higher. Analogous to condition (1), we find the condition

(2) $$s>\frac{4n}{2n^{2}-3n-1}\approx \frac{2}{n}\;\Leftrightarrow \;sn≳2$$

(Fig. 2 and Supplementary Fig. 3b), i.e. the strength of selection needs to be twice as strong or the copy number twice as large for a heterozygosity window to appear as for a replicon with regular replication. This is consistent with the finding that the decay of heterozygotes through replicon segregation is faster under random replication than under regular replication, where the heterozygote loss rates are $\eta^{\left(\text{reg}\right)}\approx \frac{1}{n}$ and $\eta^{\left(\text{ran}\right)}\approx \frac{2}{n}$ for regular and random segregation, respectively [Equations (A17) and (A20)].

With our model, the choice of the fixation threshold can influence the length of the heterozygosity window Δt ($x_{\text{thr}}=99\%$ in all results in the main text). If the initial frequency of mutant cells is very small, f≪1%, or the selective advantage not sufficiently strong, s≪4/n, the heterozygosity window length Δt is smaller with $x_{\text{thr}}=99\%$ than with a larger threshold (see Supplementary Fig. 1 for an example). In the limit $x_{\text{thr}}\to 1$, however, it converges to a size that is independent of the specific choice of *f* (see Supplementary Fig. 1 and Supplementary Information Section 1 for a mathematical proof). This shows that the appearance of the heterozygosity window is a robust phenomenon and not an artifact of our specific choices of *f* and $x_{\text{thr}}$.

### The heterozygosity window is large if the copy number is high and the selection strong

The fixation times at the phenotype and genotype levels *t*_phen_ and *t*_fix_ both increase with the replicon copy number (Fig. 3a). This is not a consequence of our choice of the initial condition, for which the initial frequency of mutant replicon copies $f_{\text{rep}}=f/n$ is smaller for higher *n*: keeping $f_{\text{rep}}$ rather than *f* constant, the fixation times are independent of *n* for small *n* and *s* where no heterozygosity window occurs but still increase with *n* otherwise (Supplementary Fig. 5).

The heterozygosity window length Δt increases with the replicon copy number *n* and—over large parts of the parameter range—with the strength of selection *s* (Figs. 2 and 3b, Supplementary Fig. 6b). For very high strength of selection *s*, the heterozygosity window length Δt is again smaller due to a decrease in the overall fixation times. If scaled with the fixation time $t_{\text{fix}}$, the size of the heterozygosity window $\Delta t/t_{\text{fix}}$ monotonically increases with *s* and eventually converges (Supplementary Fig. 4).

A mathematical analysis of the fixation process (provided in Supplementary File 1) shows that for large $x_{\text{thr}}$ (formally $x_{\text{thr}}\to 1$), the heterozygosity window length is approximately given by

(3) $$\Delta t\approx \frac{n}{1+s}\text{ln}\left(\frac{2ns}{1+s}\right)$$

for regular replication and by

(4) $$\Delta t\approx \frac{n/2}{1+s}\ln\left(\frac{ns}{1+s}\right)$$

for random replication (see Supplementary Figs. 1 and 2 for a comparison to numerical simulations). Hence, the implementation of random replication in the model leads to a reduced window size in a similar manner as a reduction in the replicon copy number by a factor of 2.

The heterozygosity window has a second dimension in addition to its length—the frequency of cells that carry the wild-type allele at the time of phenotypic fixation. This frequency, $x_{\text{wt}}(t_{\text{phen}})$, is given by the sum of the frequencies of homozygous wild-type cells $1-x_{\text{thr}}$ and of heterozygous cells $x_{\text{het}}(t_{\text{phen}})$. In Supplementary Information Section 1, we show that for large thresholds $x_{\text{thr}}$, it holds that

(5) $$x_{\text{het}}(t_{\text{phen}})\approx (r-1)\left(1-x_{\text{thr}}\right),$$

where *r* is increasing in *s* and *n* and given in Equation (S1.11). This fraction decreases with the threshold $x_{\text{thr}}$, which is expected since over time more and more cells are mutant homozygous cells. Yet, since bacterial population sizes are usually large, even a small frequency corresponds to a large number of cells. Figure 3c shows the number of heterozygotes at $t_{\text{phen}}$ for our default threshold $x_{\text{thr}}=99\%$ [cf. Supplementary Fig. 2 for a comparison of approximation (5) with numerical simulations for 2 different thresholds]. For the total fraction of cells that still carry the wild-type allele, including those that are homozygous for the wild-type allele, we obtain

(6) $$x_{\text{wt}}(t_{\text{phen}})\approx x_{\text{het}}(t_{\text{phen}})+1-x_{\text{thr}}=r(1-x_{\text{thr}}).$$

### The heterozygosity window also exists in small finite populations but is smaller

The deterministic analysis in the previous section ignores stochastic fluctuations in the genotype frequencies, reflecting the dynamics in an infinite or very large population. To account for finite population sizes, we complemented our analysis with stochastic simulations. Unlike in the deterministic model, the mutant allele can go extinct while rare, and we consider fixation times conditioned on fixation of the mutant allele. To render the results comparable to those of the deterministic model, we again define that phenotypic fixation is reached when 99% of cells are mutant cells. Similarly, the fixation at the genotype level is reached when 99% of cells are homozygous mutant cells. More precisely, since cell frequencies are subject to stochastic fluctuations and may hit the threshold several times, we consider the mean of all time points where the respective cell frequencies reach the threshold from below.

**Fig. 4.**
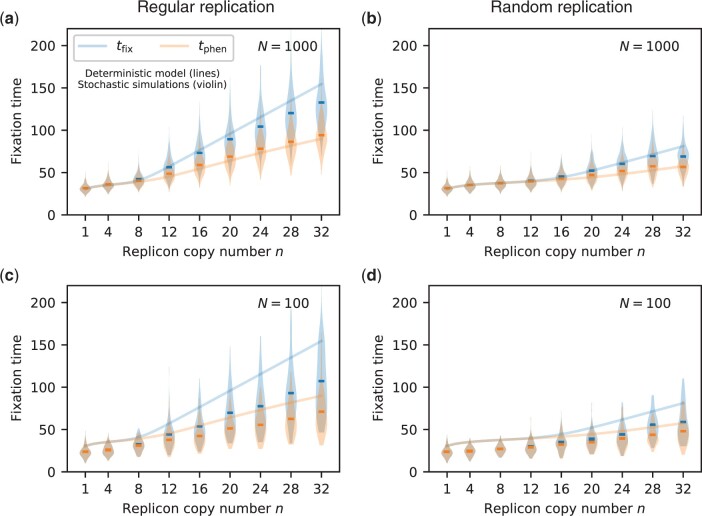
Fixation times *t*_phen_ and *t*_fix_ from stochastic simulations for selected replicon copy numbers (violin plots, mean shown as horizontal line) in comparison to results from the deterministic model (lines), *s *=* *0.3. (a, c) show results for regular replication, while (b, d) show results for random replication for population sizes N = 1000 and N = 100, respectively. To compare between stochastic and deterministic calculations, we define fixation at the phenotype level *t*_phen_ (resp. at the allele level *t*_fix)_ by the state where the frequency of mutants (resp. of mutant homozygotes) hits a threshold of 0.99. For each trajectory of the 1,000 trajectories, we determine the fixation times *t*_phen_ and *t*_fix_ by the mean of all time points at which the respective frequencies increase to the fixation threshold from below.

We find that a heterozygosity window also occurs in finite populations (Fig. 4). For small populations, the heterozygosity window is smaller than predicted by the deterministic model, especially if the replicon copy number *n* is high (Figs. 4, c and d, Supplementary Fig. 7). In finite populations, we could follow the allele dynamics up to true genotypic fixation, i.e. up to *x_n_* = 1. The window sizes that can be seen in Fig. 4 and Supplementary Fig. 7 are lower limits for the full time period during which genetic variation persists, while the population is already well adapted.

For monoploid populations (*n *=* *1), the expected fixation time of a mutant allele decreases with the population size (Kimura and Ohta 1969). Similarly, we find that the fixation of homozygous mutant cells *t*_fix_ is faster in finite populations than predicted by the deterministic model; furthermore, the time to fixation decreases with the population size (Fig. 4). The phenotypic fixation time *t*_phen,_ however, reaches a maximum for an intermediate population size (cf. the fixation times for *N *=* *1,000 with those for *N *=* *100 and those predicted by the deterministic model reflecting an infinite population in Fig. 4a and Supplementary Fig. 7).

### A heterozygosity window also exists if the cell type frequencies are in transformation–selection balance prior to adaptation

So far, we assumed a given initial frequency *f* of mutant cells where each of those cells carries one mutant copy. This corresponds, for example, to the cell-type composition after incorporating a mutant allele into the plasmid via transformation (e.g. Garoña *et al.* 2021). In natural settings, however, mutations are often present at low levels for a long time in a balance between negative selection and recurrent appearance before they become beneficial due to a shift in the environmental conditions. In that case, cells with more than one mutant replicon copy may arise before the fixation process ensues.

In the following, we therefore model 2 phases—the first one, in which the mutant allele is subject to negative selection, modeled by a reduced cell division rate 1−σ of mutant cells, and a second one, when it has turned beneficial and rises to fixation. For the first phase, we assume that the mutant allele appears in single replicon copies at a transformation rate *τ* per cell per time unit and determine the mutant cell frequencies in the equilibrium between the input of the mutant allele via transformation and loss due to negative purifying selection. At time point *t *=* *0, the mutant allele becomes beneficial, i.e. mutant cells divide at rate 1+s as in the above sections. A detailed description of the model is given in Appendix A4.

**Fig. 5.**
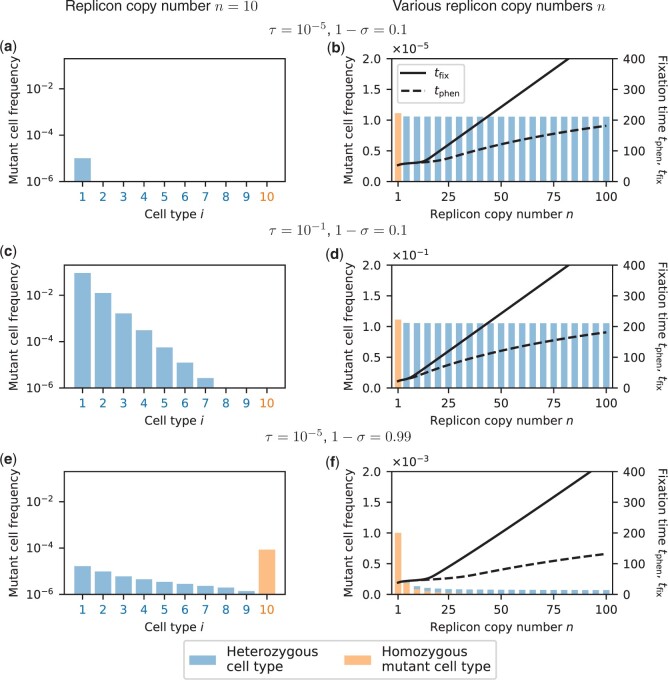
Mutant cell frequencies at transformation–selection balance for replicon copy number *n *=* *10 (a, c, e) and for various replicon copy numbers *n* (b, d, f). a, c, e) Example with *n *=* *10 replicon copies per cell. Deterministic frequencies of the different mutant cell types (number of mutant replicon copies per cell, *x*-axis) in the population. The remaining fraction of cells is wild-type homozygotes with *i *=* *0. Single mutant replicon copies enter cells at a rate *τ* and decrease the cell division rate by *σ*. Cell-type frequencies are shown on a logarithmic scale, allowing a direct comparison between various selection coefficients *σ* and transformation rates *τ*. b, d, f) Mutant cell frequencies for various replicon copy numbers *n* (*x*-axis). Bar plots show mutant frequencies on a linear scale, which allows a comparison of the composition of heterozygous (blue) and homozygous mutant cells (orange). Note that the scale differs in all 3 panels. Line plots show times until fixation at the phenotype level *t*_phen_ (dashed) and at the genotype level *t*_fix_ (solid) when the process is started at transformation–selection balance. Note that in (b), the heterozygosity window would open for smaller values of *n* if the thresholds for *t*_phen_ and *t*_fix_ were chosen closer to 1. During the fixation process, mutant cells divide at rate 1+s with *s *=* *0.3 (wild-type cells divide at rate 1). Fixation times were obtained from deterministic simulations, Equation (A11) as in Fig. 3.

Overall, we find that the general pattern of the heterozygosity window occurrence remains unchanged, irrespective of *τ* and *σ*: a window opens up for sufficiently large *n* (Fig. 5).

Strongly deleterious mutations (cell division rate 1−σ close to 0) mostly occur in heterozygous cells with few mutated replicon copies in transformation-selection balance (Fig. 5, a–d). Furthermore, the frequency of mutant cells is nearly independent of *n* (Fig. 5, b and d). For very low transformation rates, most of the mutant cells contain a single mutant replicon copy, which resembles the scenario that we considered in the above sections (Fig. 5a). For high transformation rates, cells with more than one mutant replicon copy exist, which reduces fixation times for low-copy replicons but not for high-copy replicons (compare Fig. 5, b and d).

If the strength of selection is weak (1−σ close to 1), mutant cells can persist longer in the population on average. Therefore, homozygous mutant cells can be generated and exist at transformation-selection balance for low-copy numbers (Fig. 5, e and f). For high replicon copy numbers *n*, however, too many generations would be needed for homozygous cells to emerge; thus, almost all mutant cells are heterozygous at transformation–selection balance even if selection is weak. Unlike for strong selection, the overall frequencies of mutant cells strongly decrease with *n*. Nonetheless, for high *n*, fixation times are smaller compared to the case of strong selection (Fig. 5, f and b).

### The mode of replicon segregation strongly influences the occurrence and length of a heterozygosity window

In the previous sections, we considered random segregation of replicon copies at cell division. Here, we examine the effect of alternative segregation modes on the fixation dynamics (Fig. 1). All 3 alternative modes in our model reflect a more deterministic form of replicon inheritance compared to the baseline model of random segregation.

**Fig. 6.**
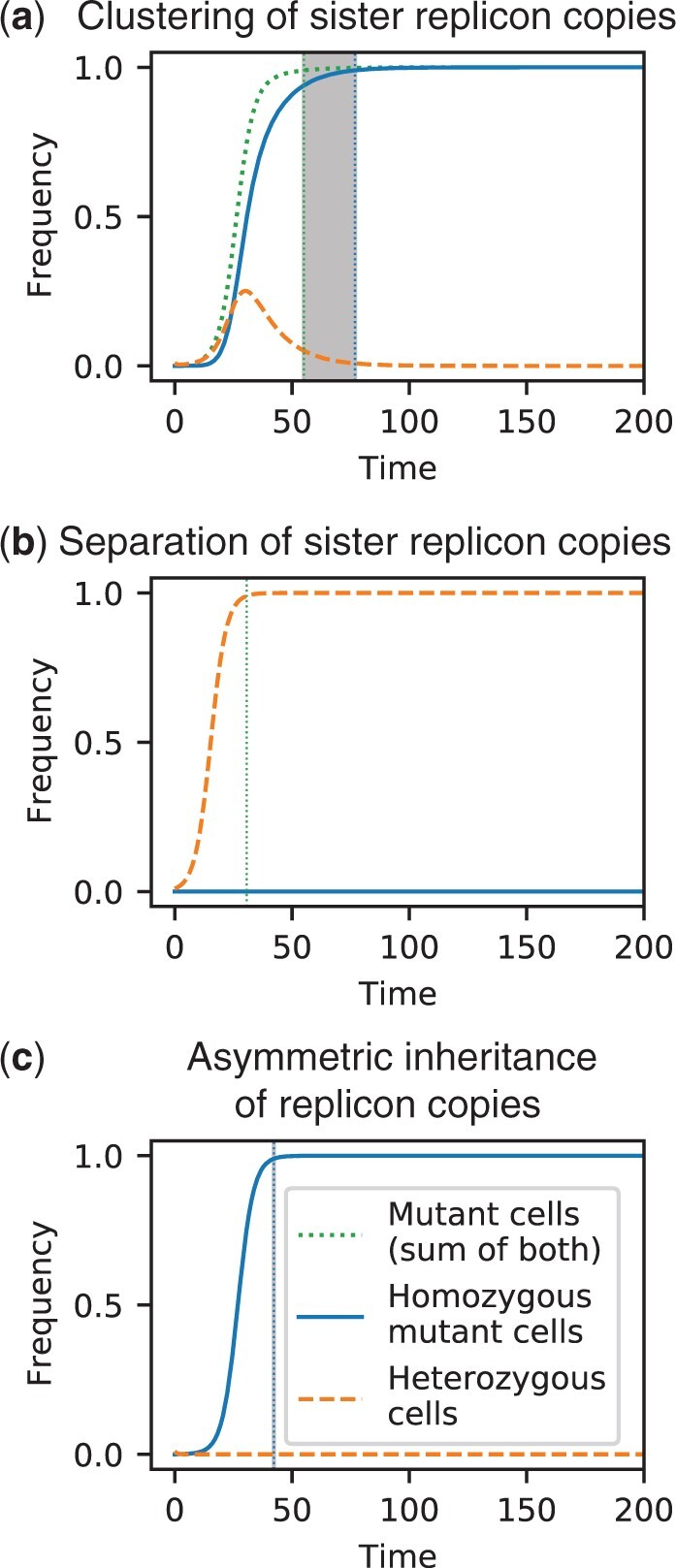
Frequency trajectories of different cell types for alternative models of replicon segregation in combination with regular replication: For (a) *clustering of sister replicon copies*, all pairs of sister replicon copies are inherited to the same daughter cell, whereas for (b) *separation of sister replicon copies*, sister replicon copies segregate into different daughter cells. c) For the mode of *asymmetric inheritance of replicon copies*, one of the daughter cells of a heterozygous cell receives all mutant replicon copies. Parameters: Replicon copy number *n *=* *32, strength of selection *s *=* *0.3, initial frequency of mutant cells with one mutant replicon copy *f *=* *0.01 (cf. Fig. 2d for the baseline model of random segregation). The time unit corresponds to the generation time of the wild type. Calculations and visualization were performed as in Fig. 2.

Notably, the *clustering of sister replicon copies* segregation mode reduces the unit of inheritance—that is, the number of segregating DNA molecules—by a factor of 2 compared to random segregation (1). The fixation dynamics under *clustering of sister replicon copies* with copy number 2*n*, therefore, resembles the resulting dynamics under random segregation with replicon copy number *n*. Both the fixation time of mutant cells and of homozygous mutant cells are reduced and the heterozygosity window is smaller if sister replicons segregate into the same cell than if they segregate independently from each other (Fig. 6a and Supplementary Fig. 8A, cf. 2d and 3a for the baseline model with random segregation). In line with our other results on random and regular replication [Equations (3) and (4)], fixation times *t*_phen_ and *t*_fix_ and the size of the heterozygosity window for *clustering of sister replicon copies* are very similar to those obtained for random replication (cf. Supplementary Figs. 6a and 8a).

Under the mode of *separation of sister replicon copies*, cells with *i* mutant replicon copies always produce 2 daughter cells of the same type *i* since every sister couple is equally divided between the 2 daughter cells. Hence, the mutation will never reach fixation at the genotype level (Fig. 6b), and in the absence of gene conversion and without deviations from the model, heterozygosity is maintained forever. The fixation dynamics at the phenotypic level are effectively reduced to the case *n *=* *1 as wild-type cells divide into wild-type cells, and mutant cells divide into mutant cells of the same type *i* (Supplementary Fig. 8b). Consequently, the fixation time of mutant cells is independent of the replicon copy number in the *separation of sister replicon copies* mode.

Last, we consider the *asymmetric inheritance of replicon copies* mode, which reflects a more extreme scenario of sister replicon clustering. Following the mode of asymmetric inheritance, heterozygous cells always divide into one heterozygote and one homozygous cell. Consequently, there is no increase in heterozygous cells, and no heterozygosity window appears (Fig. 6c). A comparison of the fixation dynamics for different replicon copy numbers shows that the fixation time increases slightly with the copy number *n* (Supplementary Fig. 8c). The reason for this increase is the smaller initial mutant replicon frequency on the allele level $f_{\text{rep}}=f/n$ for higher *n*. For a constant initial mutant frequency on the allele level $f_{\text{rep}}=f/n$, the fixation time is independent of the copy number *n* in this inheritance mode (cf. Supplementary Fig. 9).

## Discussion

To understand the consequences of polyploidy for allele dynamics in prokaryotes, we considered the fixation process of a dominant beneficial mutation on a multicopy replicon.

### Maintenance of heterozygosity on multicopy replicons

Our initial model in which replication is regular and segregation random shows that fixation times are longer on multicopy replicons than on single-copy replicons and increase with the copy number. This is generally in line with experimental and theoretical results by Ilhan *et al.* (2019), who simulated the distribution of replicon copy variants in the daughter cells and the cell composition in the next generation by binomial sampling. For large copy numbers and strong selection [see Equation (1)], we moreover find a delay between fixation at the phenotype level and fixation at the genotype level (defined here as reaching threshold frequencies), which we term “heterozygosity window.” Within the heterozygosity window, the population is phenotypically (almost) fully adapted, while genetic variation is maintained. For example, de novo evolution of antibiotic resistance would be reversible during the heterozygosity window if antibiotics are removed, and the resistance mutation has a negative fitness effect in the absence of antibiotics. Importantly, such a reversible adaptation would leave no trace in the genome. However, how easily the population can adapt to such a future change also depends on the dominance relationship between the 2 alleles in the new environment (e.g. antibiotic-free environment) and the composition of the wild-type allele carrying subpopulation, and future models are needed to assess this. In our model, no appreciable heterozygosity window emerges if selection is weak or the copy number is low. These results hold, irrespective of whether the adaptive process starts from a constant low fraction of mutant cells with one mutant copy each, a constant low fraction of mutant replicon copies (with no more than one copy in each cell), or from transformation–selection balance (Figs. 3a and 5).

The existence of a heterozygosity window can be understood in the following way: if selection is strong, heterozygous cells quickly rise in frequency. At the same time, if the copy number is large, homozygous cells emerge only slowly, and the mutant cells become (nearly) fixed before mutant homozygotes dominate the population. From this point on, the process is selectively neutral except for homozygous wild types that are generated during cell division. The emergence of such wild-type cells is rare for high replicon copy numbers such that the fixation of homozygous mutant cells is slow. If selection is weak or the copy number low, homozygous mutant cells are generated early in the adaptive process. They quickly rise in frequency since all daughter cells of homozygous cells are themselves homozygous. Heterozygous cells, in contrast, rise only little or not at all since they segregate too many offspring into the homozygous classes. In that scenario, the phenotypic fixation coincides with genotypic fixation.

Most of our analysis relies on a deterministic model for the genotype frequencies in the population. Stochastic simulations show that the heterozygosity window is smaller than predicted by the deterministic model if the population size is small, but qualitatively, the results also hold in small finite populations. In addition, we find that the phenotypic fixation time has a maximum for intermediate population sizes.

In this study, we focused on dominant mutant alleles, where a heterozygosity window is expected to be most prominent. Mutations with a high degree of dominance have been detected, for example, in plasmid-carried genes coding for FolA, a key enzyme in the folate metabolism. Cells that are heterozygous or homozygous for this mutation have similar levels of resistance to trimethoprim (Rodríguez-Beltrán *et al.* 2020). For a recessive mutant allele, heterozygous cells have no selective advantage over wild-type cells, and therefore do not rise in frequency by natural selection. Once homozygous mutant cells finally emerge, they rapidly rise to fixation (Supplementary Fig. 10a). The dynamics of mutant alleles of intermediate dominance (i.e. the cell fitness increases with the frequency of the mutant allele) are positioned between these 2 extremes, and the effects leading to a heterozygosity window are less pronounced than for a dominant mutation (Supplementary Fig. 10b). This is likely similar for alleles with a gene dosage effect (i.e. where the phenotype depends on the number of mutant replicon copies).

Experimentally, an initial rise and subsequent decline of heterozygotes and a heterozygosity window have been observed in invasion experiments of a beneficial allele on a multicopy plasmid (see Fig. 3 in Rodríguez-Beltrán *et al.* 2018). Complimentary computer simulations show that heterozygosity can be maintained for many generations if the selection pressures for the 2 alleles rapidly alternate. In these simulations, Rodríguez-Beltrán *et al.* (2018) made the simplifying assumption that all heterozygous cells contain the 2 plasmid variants in equal proportions, i.e. there are only 3 cell types—the 2 homozygous types and heterozygous cells. Assuming that plasmid copies segregate to the daughter cells with probability 1/2, the probability that a heterozygous cell forms 2 homozygous cells at cell division is $2^{1-n}$ (using our model formulation, where plasmid copies are replicated prior to cell division). Using this assumption, $2^{1-n}$ is the heterozygote loss rate in the absence of selection [see A.3, Equation (A17)]. Our analysis, which explicitly considers heterozygous cells with different compositions of the replicon pool, shows that the heterozygote loss rate is more accurately described by $\eta^{\left(\mathrm{reg}\right)}=\frac{1}{n-1/2}\approx \frac{1}{n}$ for regular replication [see Equation (A.17)]. Comparing these 2 results shows that the approximation in Rodríguez-Beltrán *et al.* (2018) underestimates the loss rate, especially for high replicon copy numbers *n*. Similar to our model, Novick and Hoppensteadt (1978) calculated the decrease in the proportion of heterozygous cells per generation in a geometrically growing population as $\frac{1}{2n-1}\approx \frac{1}{2n}$ for plasmid copies undergoing regular replication. The difference of a factor 1/2 between the loss rate in our model and the loss rate in Novick and Hoppensteadt (1978) is due to the different population dynamics in the 2 models (constant population size vs geometric growth).

It is interesting to compare fixation on a multicopy replicon in an asexually reproducing population to the fixation of a beneficial allele in a diploid sexually reproducing population: in the latter, there is also a heterozygosity window for dominant (but not for recessive) alleles (Supplementary Fig. 11). The underlying dynamics are, however, very different since the generation of homozygous individuals requires mating of heterozygous individuals, and mating of the 2 homozygous types regenerates heterozygous individuals.

### The effects of the segregation and replication modes

The modes of replication and segregation depend on the respective replicon type and on the species. In our study, we consider several fundamental modes for both processes. In the future, the model can be tailored to accurately describe the details of specific systems. We did not consider horizontal transfer of replicon copies, which occurs for many types of plasmids. For strong selection and low rates of transfer, the effect of horizontal transfer on the size of the heterozygosity window is presumably minor but would need to be assessed in future models.

For most of our analysis, we assumed that each replicon copy is replicated exactly once prior to cell division (regular replication). We considered 4 modes of replicon copy segregation. In all modes considered, both daughter cells inherit the same number of replicon copies. The segregation mode affects the allele distribution in the daughter cells and hence the maintenance or loss of intracellular genetic variation. Variability in the replicon copy number would affect the size of the window. Yet, at least for high copy numbers with a prominent heterozygosity window, we suspect that variability would not alter the main conclusions since the relative deviation between the copy numbers of the 2 daughter cells is low for high copy numbers.

Under the mode *separation of sister replicon copies*, the heterozygosity window is infinitely long, i.e. heterozygosity is maintained forever. The complete opposite dynamics occur under the mode of *asymmetric inheritance of replicon copies* where one of the daughter cells inherits the maximum possible number of mutant copies. In that case, heterozygous cells do not increase in frequency since cell division of heterozygotes leads to one heterozygous cell and one homozygous cell. Thus, in monoploid bacteria that become effectively polyploid during fast growth due to multifork replication, heterozygosity will rapidly decrease, and no heterozygosity window arises. The results obtained applying the modes of *random segregation* and of *clustering of sister replicon copies* lie between perfect separation of copies and asymmetric inheritance. In these modes, heterozygous subpopulations can rise transiently, given that the replicon copy number and the strength of selection are sufficiently high, and a heterozygosity window opens up. Since the replication of plasmids and likely also of some types of chromosomes is better described by random than by regular replication of copies, we also modeled the replication mode *random replication* (in combination with random segregation of replicon copies). In that case, the heterozygosity window is also present but smaller than under regular replication. Notably, our results show that it is approximately as large as for a regularly replicating replicon with a copy number n/2. For example, for a plasmid with copy number n≈20 [as in San Millan *et al.* (2016)] that is undergoing random replication and segregation, there is only a delay of a few (wild-type) generations between phenotypic and genotypic fixation even if selection for the mutant allele is strong (Supplementary Fig. 6). For a replicon undergoing regular replication, there would be a delay of around 10–40 generations with *s *=* *0.1–0.3 (Fig. 3). Fitness effects of this order of magnitude have been found in *E. coli* in various contexts including for several mutations during long-term adaptation (Rozen *et al.* 2002; Ferenci 2008; Couce *et al.* 2022). For *Pseudomonas fluorescens* evolving in a stressful environment, the selection coefficients of mutations were found to be even an order of magnitude higher (Barrett *et al.* 2006).

Our modeling framework could be applied to support experimental studies in polyploid species. The mode of chromosome segregation differs between prokaryotic species and is not always well understood. Following the fate of heterozygous cells has been used as one approach to gain insights into the segregation patterns (e.g. Pulakat *et al.* 1998; Suh *et al.* 2000; Tobiason and Seifert 2010; Li 2019). With our modeling framework, we can make quantitative predictions on the maintenance of heterozygosity and the time to loss or fixation of a marker. This makes it possible to test on a quantitative basis which segregation patterns are compatible with experimental observations and thus to better understand which conclusions can and cannot be drawn. A second application of our model concerns genetic engineering. Genetic engineering is known to be difficult in highly polyploid species such as *Synechocystis PCC 6803*, which carries approximately 60 chromosome copies per cell (Griese *et al.* 2011). To incorporate an allele into all chromosome copies, positive selection for this allele needs to be applied for a large number of generations, that is, until genotypic fixation. If selection is released too early, reversion to the wild type may occur. Our model allows us to estimate the required number of generations in advance. Especially, it provides us with an estimate for the number of generations for which selection needs to be maintained once the mutant phenotypic is close to fixation, the latter being detectable in the lab. There is, however, a caveat with applying our current model to experimental studies: we here assume a constant population size, while in most experiments, the population size drastically changes in the alternation between exponential growth and population bottlenecks. This very likely affects the size of the heterozygosity window. Yet, our model can be readily adjusted to account for such population dynamics, e.g. by replacing the Moran model by a birth–death model and including bottlenecks at regular intervals.

In this study, we focused on the dynamics of alleles on multicopy replicons in prokaryotes. Similar dynamics and questions arise for polyploid eukaryotic cells. Mitotic cell division leads to a division of sister replicon copies. Some replicon types in eukaryotes, however, do not undergo mitosis, for example, chromosomes in the somatic macronucleus of ciliates (Morgens and Cavalcanti 2015), nuclear extra-chromosomal DNA (ecDNA) in tumor cells (Bailey *et al.* 2020), mitochondria, and other organelles (Lightowlers *et al.* 1997; Stewart and Chinnery 2015; Ramsey and Mandel 2019). Specifically, heteroplasmy is known to occur in mitochondria, and the spread of mutations in mitochondrial DNA through replication of mutated copies and segregation at cell division within an organism or across generations is highly relevant in the context of disease development (Lawless *et al.* 2020). In all the above cases—macronucleus of ciliates, ecDNA, mitochondria, and other organelles—the segregation of replicon copies is random or at least partially so, and related modeling approaches have been applied to either study variation in the number of replicon copies or of genetic variants (e.g. Kimura 1957; Morgens and Cavalcanti 2015; Pichugin *et al.* 2019; Lawless *et al.* 2020). Our results could thus also be of interest for multicopy replicons in eukaryotes.

### Conclusion

Heterozygosity is commonly considered in diploid sexually reproducing organisms. Prokaryotic cells can be heterozygous as well if they harbor a multicopy replicon, i.e. a polyploid chromosome or a multicopy plasmid. The present work demonstrates that heterozygosity of multicopy replicons, hence genetic variation, can be maintained for extended periods of time—the heterozygosity window—during the fixation process of a dominant beneficial mutation.

## Supplementary Material

iyac121_File_S1

## Data Availability

The authors state that all data necessary for confirming the conclusions presented in the article are represented fully within the article. Supplementary information and figures can be found in Supplementary File 1. The simulation code and the scripts used for computer algebra are available on figshare (Supplementary File 2, http://doi.org/10.25386/genetics.20264070). Supplemental material is available at *GENETICS* online.

## Appendix A

### A1. Mathematical formulation of the model and stochastic computer simulations

We describe the dynamics of the system by a state vector $N(t)=\left(N_{0}(t),N_{1}(t),\ldots ,N_{n}(t)\right)$, where $N_{i}(t)$ denotes the number of cells with *i* mutant replicon copies (“cells of type *i*”). For all times *t*, the total number of cells $N=\sum_{i=0}^{n}N_{i}$ is constant, whereas the relative abundances of the different types may change. The number of cells of any type *i* can be altered either by cell division or by cell death. Cell death occurs by removal of a randomly chosen cell from the population right after cell division so that the total population size remains constant.

The rate at which a cell of type *i* divides into daughter cells of type (*j*_1_, *j*_2_; ordered pair) is given by $N_{i}\lambda_{i}p_{i\to j_{1}j_{2}}$, where $p_{i\to j_{1}j_{2}}$ denotes the probability that cell division leads to daughter cells of type *j*_1_ (first daughter cell) and *j*_2_ (second daughter cell). The probability distribution $p_{i\to j_{1}j_{2}}$ depends on the mode of replication and the mode of segregation and will be derived below for the various replication and segregation modes shown in Fig. 1. The probability that a cell of type *l* is replaced following division of an *i*-type cell is given by $\nu_{l}=N_{l}/N$ if l≠i and $\nu_{l}=(N_{l}-1)/N$ if *l *=* i*. Thus, cell division events that increment the number of cells of type *j*_1_ and *j*_2_ (new daughter cells) and decrement the number of cells of type *i* (dividing cell) and of type *l* (replaced cell) occur at rate $N_{i}\lambda_{i}p_{i\to j_{1}j_{2}}\nu_{l}$. It should be noted that some or all of the cell types $j_{1},j_{2},i,l$ may be identical. Cell division events that do not change the state vector **N** can be omitted in the simulations and when deriving the deterministic dynamics.

We now derive the probability distributions $p_{i\to j_{1}j_{2}}$ for the different modes of replication and segregation. For *regular replication*, each replicon copy is duplicated, resulting in exactly k=2i mutated copies just before cell division of a type-*i* cell. For *random replication*, *k* is a random number. The successive replication of copies before cell division corresponds to a Pólya urn model (Eggenberger and Pólya 1923; Mahmoud 2008), and the probability to have *k* mutated copies before cell division is given by

(A1) $$p^{\left(\text{succ}\right)}(k;i,n):=\left(\begin{array}{c} n\\ k-i \end{array}\right)\frac{B(k,2n-k)}{B(i,n-i)}$$

for i+n≥k≥i and zero otherwise, in case of heterozygous types 0<i<n. *B* denotes the Beta-function, where, for positive integers,

(A2) $$B(x,y)=\frac{(x-1)!(y-1)!}{(x+y-1)!}.$$

For homozygous types *i *=* *0, *i *=* n*, we have $p^{\left(\text{succ}\right)}(k;i,n):=\delta_{k,2i}$, where $\delta_{k,2i}$ denotes Kronecker’s delta.

Mutant and wild-type replicon copies are distributed to the daughter cells according to the chosen mode of segregation. In all of them, each daughter cell receives exactly *n* replicon copies in total. In the case of *random segregation*, the probability that a cell containing *k* mutant copies just before division produces 2 daughter cells of types (*j*_1_, *j*_2_) is given by

(A3) $$\frac{\left(\begin{array}{c} k\\ j_{1} \end{array}\right)\left(\begin{array}{c} 2n-k\\ n-j_{1} \end{array}\right)}{\left(\begin{array}{c} 2n\\ n \end{array}\right)}\delta_{k,j_{1}+j_{2}},$$

where $\delta_{k,j_{1}+j_{2}}$ denotes Kronecker’s delta. If we combine this term with k=2i for *regular replication* (reg) or with the probability distribution for *random replication* (ran), we obtain

(A4) $$p_{i\to j_{1}j_{2}}^{\left(\text{reg}\right)}=\frac{\left(\begin{array}{c} 2i\\ j_{1} \end{array}\right)\left(\begin{array}{c} 2n-2i\\ n-j_{1} \end{array}\right)}{\left(\begin{array}{c} 2n\\ n \end{array}\right)}\delta_{2i,j_{1}+j_{2}}$$

(A5) $$p_{i\to j_{1}j_{2}}^{\left(\text{ran}\right)}=\sum_{k=i}^{i+n}p^{\left(\text{succ}\right)}(k;i,n)\frac{\left(\begin{array}{c} k\\ j_{1} \end{array}\right)\left(\begin{array}{c} 2n-k\\ n-j_{1} \end{array}\right)}{\left(\begin{array}{c} 2n\\ n \end{array}\right)}\delta_{k,j_{1}+j_{2}}.$$

For the segregation modes, where sister replicons are clustered (ii) or separated (iii) to different daughter cells we only consider *regular replication* since *random replication* does not allow defining unique pairs of sister replicons. Similarly, we do not consider *random replication* for the segregation mode (iv) *asymmetric inheritance of replicon copies* since replicating one copy 2 times would violate our assumption of equal copy numbers in both daughter cells: For *asymmetric inheritance of replicon copies*, the 2 replicon copies that form the oldest link in genealogy are segregated to distinct daughter cells together with all their younger sisters (see below). Therefore, it is needed that each copy is duplicated (regular replication) once so that both daughter cells receive *n* replicon copies.

In the case of *clustered segregation of sister replicons* (clu) combined with *regular replication*, we need to consider the random distribution of *i* pairs of mutant replicon copies instead of 2*i* individual mutant replicon copies (cf. Equation A4). The probability that an *i*-type cell with *i* pairs of mutant copies before cell division produces 2 daughter cells with $\left(j_{1}/2,j_{2}/2\right)$ mutant couples, respectively, can be derived by replacing 2i→i, 2n→n, and $2j_{m}\to j_{m}$, *m *=* *1, 2 in Equation (A4). We obtain

(A6) $$p_{i\to j_{1}j_{2}}^{\left(\text{cou}\right)}=\{\begin{array}{cc} \frac{\left(\begin{array}{c} i\\ j_{1}/2 \end{array}\right)\left(\begin{array}{c} n-i\\ n/2-j_{1}/2 \end{array}\right)}{\left(\begin{array}{c} n\\ n/2 \end{array}\right)}\delta_{2i,j_{1}+j_{2}} & \;\;\text{for}\;\;j_{1}\;\text{even},\\ 0 & \;\;\text{otherwise}. \end{array}$$

For this mode, we need to restrict the replicon copy number to even numbers *n*.

For the mode of *separation of sister replicon copies* (sep), reproduction of *i*-type cells produces only daughter cells of type $j_{1}=j_{2}=i$. Thus, we have

(A7) $$p_{i\to j_{1}j_{2}}^{\left(\text{sep}\right)}=\delta_{i,j_{1}}\delta_{i,j_{2}}.$$

For the mode of *asymmetric inheritance of replicon copies* (asy), a heterozygous cell of type *i*, where 0<i<n/2, divides into one daughter cell with twice the number of mutated copies than the parental cell and one daughter cell with only wild-type copies, i.e. $j_{1}=2i$ and $j_{2}=0$. (From this, it follows that there are no heterozygous cells with i>n/2.) Homozygous cells divide into 2 homozygous daughter cells of the same type. Therefore, the probability that an *i*-type cell produces daughter cells (*j*_1_, *j*_2_) is given by

(A8) $$p_{i\to j_{1}j_{2}}^{\left(\text{asy}\right)}=\{\begin{array}{cc} \delta_{2i,j_{1}}\delta_{0,j_{2}} & \;\;\text{if}\;\;0<i<n/2,\\ \delta_{n,j_{1}}\delta_{n,j_{2}} & \;\;\text{if}\;\;i=n,\\ \delta_{0,j_{1}}\delta_{0,j_{2}} & \;\;\text{if}\;\;i=0. \end{array}$$

We perform stochastic computer simulations using the Gillespie algorithm (Gillespie 1976), which implements the models exactly. The simulation code is written in the Python programming language (Supplementary File 2).

### A2. Derivation of the deterministic dynamics

The derivation of the deterministic dynamics is identical to Santer and Uecker (2020). We recapitulate it here such that the article is self-contained.

To obtain the deterministic dynamics, we look at all events that alter the number of cells *N_j_* of a distinct type *j*. The following events can alter *N_j_*: (1) Cell division of *j*-type cells, which occurs at rate $N_{j}\lambda_{j}$ and reduces *N_j_* by 1. (2) When cells of any type *i* divide, they may produce *m *=* *1 or *m *=* *2 daughter cells of type *j*, which increases *N_j_* by *m*, with probability

(A9) $$p_{i\to j}^{\left(m\right)}:=\{\begin{array}{cc} \sum_{\begin{array}{c} j\neq j\prime \\ j\prime =0 \end{array}}^{n}p_{i\to jj\prime }+p_{i\to j\prime j} & \text{for}\;m=1,\\ p_{i\to jj} & \text{for}\;m=2. \end{array}$$

(3) Replacement of a randomly chosen cell of type *j*, which reduces *N_j_* by 1. All combinations of these 3 events with the corresponding rates are listed in Table A1. Putting all those terms together, we obtain

(A10) $$\begin{array}{c} {\dot{N}}_{j}=N_{j}\lambda_{j}p_{j\to j}^{\left(2\right)}(1-\frac{N_{j}-1}{N-1})-N_{j}\lambda_{j}p_{j\to j}^{\left(1\right)}\frac{N_{j}-1}{N-1}\\ -2N_{j}\lambda_{j}(1-p_{j\to j}^{\left(1\right)}-p_{j\to j}^{\left(2\right)})\frac{N_{j}-1}{N-1}-N_{j}\lambda_{j}(1-p_{j\to j}^{\left(1\right)}-p_{j\to j}^{\left(2\right)})(1-\frac{N_{j}-1}{N-1})\\ +\sum_{\overset{i=0}{i\neq j}}^{n}N_{i}\lambda_{i}p_{i\to j}^{\left(2\right)}\frac{N_{j}}{N-1}+2N_{i}\lambda_{i}p_{i\to j}^{\left(2\right)}(1-\frac{N_{j}}{N-1})\\ +N_{i}\lambda_{i}p_{i\to j}^{\left(1\right)}(1-\frac{N_{j}}{N-1})-N_{i}\lambda_{i}(1-p_{j\to j}^{\left(1\right)}-p_{j\to j}^{\left(2\right)})\frac{N_{j}}{N-1}\\ \approx \sum_{i=0}^{n}N_{i}\lambda_{i}(2p_{i\to j}^{\left(2\right)}+p_{i\to j}^{\left(1\right)}-\frac{N_{j}}{N})-N_{j}\lambda_{j}, \end{array}$$

where we have used N−1≈N (Santer and Uecker 2020). The deterministic dynamics of the system is obtained by simultaneously integrating all *n *+* *1 equations for the cell-type frequencies $x_{j}=\frac{N_{j}}{N},\;j=0,\ldots ,n$:

(A11) $${\dot{x}}_{j}=\sum_{i=0}^{n}\{x_{i}\lambda_{i}(\underset{=:m_{i\to j}}{\underset{︸}{2p_{i\to j}^{\left(2\right)}+p_{i\to j}^{\left(1\right)}}}-x_{j})\}-x_{j}\lambda_{j},$$

where we introduced $m_{i\to j}$ as the expected number of *j*-type cells produced at division of an *i*-type cell used below. We use Python for all numerical simulations and the solve_ivp function implementing an implicit Runge–Kutta method (option “Radau”) from the SciPy Package (Virtanen *et al.* 2020) for the integration of (A11).

### A3. Mathematical derivation for the conditions under which a heterozygosity window exists under random segregation

We here derive the criteria (1) and (2) for observing a heterozygosity window given that the initial frequency of mutant cells *f* is small. By definition, a heterozygosity window occurs if there are still heterozygous cells present at the time of fixation *t*_phen_ at the phenotype level. This may simply happen if the heterozygous cells that were present at time *t *=* *0 have not fully decayed yet. This leads to a very small window though. A more relevant heterozygosity window occurs, if the heterozygotes initially increase in frequency during the fixation process (Fig. 2). This observation builds the basis of our approximation.

For the frequency of heterozygous cells, i.e. 0<j<n, we derive from Equation (A11)

(A12) $$\begin{array}{l} {\dot{x}}_{j}=\sum_{i=0}^{n}x_{i}\lambda_{i}(m_{i\to j}-x_{j})-x_{j}\lambda_{j}\\ =-\lambda_{0}x_{0}x_{j}-\lambda_{n}x_{n}x_{j}+\sum_{i=1}^{n-1}x_{i}\lambda_{i}(m_{i\to j}-x_{j})-x_{j}\lambda_{j}\\ \approx -\lambda_{0}x_{j}-\lambda_{j}x_{j}+\sum_{i=1}^{n-1}\lambda_{i}m_{i\to j}x_{i}\\ =-x_{j}-(1+s)x_{j}+\sum_{i=1}^{n-1}\left(1+s\right)m_{i\to j}x_{i}\\ =\sum_{i=1}^{n-1}((1+s)(m_{i\to j}-\delta_{ji})-\delta_{ji})x_{i}, \end{array}$$

where *δ_ji_* denotes Kronecker’s Delta. Here, we have neglected quadratic terms of mutant cell frequencies $x_{i}x_{j}$, where *i *>* *0 and *j *>* *0, as mutant cell frequencies are low at early time points in the fixation process.

We choose the initial mutant frequency *f* sufficiently low so that the relative frequencies of heterozygote types equilibrate quickly compared to the time it takes for mutant cells to reach high frequencies in the population. The frequencies of heterozygote types then take approximately the form of the right eigenvector corresponding to the leading eigenvalue of the matrix ${\left(m_{i\to j}-\delta_{ji}\right)}_{ji\in \{1,\ldots ,n-1\}}$ [see Equation (A12)]. In the following, we denote by *x_i_* the frequencies of cells of type *i* at such an early time *t* assuming that the relative heterozygous frequencies are in equilibrium.

If replication is regular, we have $m_{i\to j}=2\left(\begin{array}{c} 2i\\ j \end{array}\right)/\left(\left(\begin{array}{c} 2n-2i\\ n-j \end{array}\right)\left(\begin{array}{c} 2n\\ n \end{array}\right)\right)$ [see Equations (A4) and (A9)]. The dominant eigenvalue of the matrix ${\left(m_{i\to j}-\delta_{ij}\right)}_{ji\in \{1,\ldots ,n-1\}}$ for regular replication can be calculated explicitily as

(A13) $$\xi =\frac{2n-3}{2n-1}$$

(Novick and Hoppensteadt 1978, $\lambda^{*}$ in their Equation (2) is the dominant eigenvalue of the matrix ${\left(m_{i\to j}/2\right)}_{i,j\in \{1,\ldots ,n-1\}}$). Since $\left(x_{1},\ldots ,x_{n-1}\right)$ is the eigenvector corresponding to *ξ*, we obtain for the time derivative of the frequency of all heterozygous cells $x_{\text{het}}:=\sum_{j=1}^{n-1}x_{j}$:

(A14a) $$\begin{array}{l} {\dot{x}}_{\text{het}}=\sum_{j=1}^{n-1}{\dot{x}}_{j}\\ \approx \sum_{j=1}^{n-1}\sum_{i=1}^{n-1}((1+s)(m_{i\to j}-\delta_{ij})-\delta_{ij})x_{i}\\ =\sum_{j=1}^{n-1}((1+s)\xi -1)x_{j}\\ =((1+s)\xi -1)x_{\text{het}} \end{array}$$

(A14b) $$=((1+s)\frac{2n-3}{2n-1}-1)x_{\text{het}}.$$

Thus, heterozygous cells increase in frequency early in the fixation process if

(A15) $$\begin{array}{l} (1+s)\xi -1>0\\ \Leftrightarrow s>\frac{1}{n-3/2}\overset{n\gg 1}{\approx }\frac{1}{n}. \end{array}$$

If there is no selection, i.e. *s *=* *0, we have

(A16) $$\begin{array}{l} {\dot{x}}_{j}=\sum_{i=0}^{n}x_{i}(m_{i\to j}-x_{j})-x_{j}\lambda_{j}\\ =-x_{j}+\sum_{i=1}^{n-1}x_{i}m_{i\to j}-x_{j}\\ =\sum_{i=1}^{n-1}((m_{i\to j}-\delta_{ji})-\delta_{ji})x_{i}. \end{array}$$

(The final expression is the same as in Equation (A12), but the derivation does not require any approximation if *s *=* *0.) Analogous to Equation (A14b), we then obtain

(A17) $$\begin{array}{l} {\dot{x}}_{\text{het}}=-\frac{1}{n-1/2}x_{\text{het}}\\ =:-\eta^{\left(\text{reg}\right)}x_{\text{het}}, \end{array}$$

where $\eta^{\left(\text{reg}\right)}$ can be interpreted as the heterozygote loss rate.

For random replication, the dominant eigenvalue of the matrix ${\left(m_{i\to j}-\delta_{ij}\right)}_{ji\in \{1,\ldots ,n-1\}}$ can be calculated as

(A18) $$\xi =\frac{(2n-3)n-1}{2n^{2}+n-1}$$

(Novick and Hoppensteadt 1978). For this mode, we obtain in an analogous manner the criterion for the heterozygosity window

(A19) $$s>\frac{4n}{2n^{2}-3n-1}\overset{n\gg 1}{\approx }\frac{2}{n}.$$

and the heterozygote loss rate

(A20) $$\eta^{\left(\text{ran}\right)}=-\frac{2n}{n^{2}+\frac{n}{2}-1}\overset{n\gg 1}{\approx }\frac{2}{n}.$$

In a simpler model used by Rodríguez-Beltrán *et al.* (2018), where heterozygous cells always contain an equal proportion of mutant and wild-type copies, the dynamics of heterozygous cells without selection and at low heterozygote frequencies can be described analogous to Equation (A12) by

$${\dot{x}}_{\text{het}}=(p_{\text{het}\to \text{het}}-2)x_{\text{het}},$$

where $p_{\text{het}\to \text{het}}=2(1-2^{1-n})$ denotes the expected number of heterozygous cells created at cell division of a heterozygote. Note that $2^{1-n}$ is the probability that 2 homozygous cells are formed at cell division of a heterozygous cell (Rodríguez-Beltrán *et al.* 2018). This leads to

(A21) $${\dot{x}}_{\text{het}}=-2^{1-n}x_{\text{het}},$$

where $2^{1-n}$ is the heterozygote loss rate under this simpler model. Here, the heterozygote loss rate decreases exponentially with the replicon copy number *n*, whereas in our model, which considers different compositions of mutant and wild-type copies, the loss rate scales with 1/n.

### A4. Cell-type frequencies at transformation–selection balance

In this section, we derive the population composition at transformation–selection balance. We assume that at every transformation event, one replicon copy in the recipient cell is replaced. Mutant copies are integrated at per cell rate *τ*. For a cell with *j* mutant replicon copies, the probability that a wild-type copy is replaced is given by $\frac{n-j}{n}$. This changes its cell type j→j+1. Mutant cells of type *j *>* *0 have a reduced reproduction rate 1−σ<1, i.e. they are negatively selected. The transformation–selection balance reflects the state at which the input of the mutant variant through transformation and the selection outweigh each other. The time derivatives of the cell-type abundances ${\dot{N}}_{j}$ are given by

$${\dot{N}}_{j}\approx \{\begin{array}{ll} \sum_{i=0}^{n}N_{i}\lambda_{i}(m_{i\to 0}-x_{0})-N_{0}\lambda_{0}-N_{0}\tau & \;\text{for}\;i=0,\\ \sum_{i=0}^{n}N_{i}\lambda_{i}(m_{i\to j}-x_{j})-N_{j}\lambda_{j}+\tau (N_{j-1}\frac{n-(j-1)}{n}-N_{j}\frac{n-j}{n}) & \;\text{for}\;0<j\leq n. \end{array}$$

The time derivatives of the relative frequencies $x_{i}=N_{i}/N$ are thus given by

(A22) $${\dot{x}}_{j}=\sum_{i=0}^{n}x_{i}\lambda_{i}(m_{i\to j}-x_{j})-x_{j}\lambda_{j}+\tau \eta_{j}$$

with $\eta_{j}=(x_{j-1}-x_{j})(1-\frac{j}{n})+\frac{x_{j-1}}{n}$ for *i *>* *0 and $\eta_{0}=-x_{0}$. Finding the equilibrium $\left(x_{0},\ldots ,x_{n}\right)$ where ${\dot{x}}_{j}=0$ for all *j* was performed numerically by simultaneously integrating *x_j_* for all *j* until ${\dot{x}}_{j}/x_{j}<10^{-8}$.

**Table A1.** Events that involve cells of type *j*.

Parental cell type *i*	Daughter cell types	Cell type of replaced cell	Change in *N_j_*	Rate of reaction
*j*	*j*, *j*	*j*	0	–
*j*	*j*, *j*	≠j	+1	$N_{j}\lambda_{j}p_{j\to j}^{\left(2\right)}\left(1-\frac{N_{j}-1}{N-1}\right)$
*j*	j,≠j	*j*	–1	$N_{j}\lambda_{j}p_{j\to j}^{\left(1\right)}\frac{N_{j}-1}{N-1}$
*j*	j,≠j	≠j	0	–
*j*	≠j, ≠j	*j*	–2	$N_{j}\lambda_{j}(1-p_{j\to j}^{\left(1\right)}-p_{j\to j}^{\left(2\right)})\frac{N_{j}-1}{N-1}$
*j*	≠j, ≠j	≠j	–1	$N_{j}\lambda_{j}(1-p_{j\to j}^{\left(1\right)}-p_{j\to j}^{\left(2\right)})\left(1-\frac{N_{j}-1}{N-1}\right)$
≠j	*j*, *j*	*j*	+1	$N_{i}\lambda_{i}p_{i\to j}^{\left(2\right)}\frac{N_{j}}{N-1}$
≠j	*j*, *j*	≠j	+2	$N_{i}\lambda_{i}p_{i\to j}^{\left(2\right)}\left(1-\frac{N_{j}}{N-1}\right)$
≠j	j,≠j	*j*	0	–
≠j	j,≠j	≠j	+1	$N_{i}\lambda_{i}p_{i\to j}^{\left(1\right)}\left(1-\frac{N_{j}}{N-1}\right)$
≠j	≠j, ≠j	*j*	–1	$N_{i}\lambda_{i}(1-p_{i\to j}^{\left(1\right)}-p_{i\to j}^{\left(2\right)})\frac{N_{j}}{N-1}$
≠j	≠j, ≠j	≠j	0	–

A parental cell of type *i* produces 2 daughter cells of type {j,j′} replacing the parental cell and one randomly chosen cell of type *l*. Rates of the events are obtained by the product of cell division rates and the probability that a *j*-type cell is replaced. j,≠j denotes that one of the 2 daughter cells is of type *j* and the other is not of type *j* regardless of the order.
