# Supplementary material for: Fixation dynamics of beneficial alleles in prokaryotic polyploid chromosomes and plasmids
Source: Genetics. 2022 Aug 12;222(2):iyac121. doi: 10.1093/genetics/iyac121 (PMC9526072; doi:10.1093/genetics/iyac121)
Supplement: iyac121_File_S1 [file iyac121_file_s1.pdf]

Fixation dynamics of beneficial alleles in prokaryotic polyploid chromosomes and plasmids
Supplementary Information
File S1

Mario Santer[1], Anne Kupczok[2,3], Tal Dagan[2], and Hildegard Uecker[1]

[1]Research group Stochastic Evolutionary Dynamics, Department of Evolutionary Theory, Max Planck Institute for Evolutionary Biology, Plön, Germany
[2]Institute of General Microbiology, Kiel University, Kiel, Germany
[3]Bioinformatics group, Department of Plant Sciences, Wageningen University & Research, Wageningen, Netherlands

S1: The mathematics and convergence of the heterozygosity window

In the following, we derive an analytical approximation for the solution of cell-type frequencies over time for the assumption that the initial frequency f is very small, similar to Eq. (A.12) in section A.3. Here, we only consider the mode of random segregation. Later on we use this solution to proof the convergence of the heterozygosity window in the limit of the threshold $x_{\text{thr}} \to 1$ as discussed in the main text.

As in Appendix A.3, we choose the initial frequency f sufficiently low such that the relative frequencies of heterozygotes $\chi_j := \frac{x_j}{x_1+\cdots+x_{n-1}}$ equilibrate at a timescale that is short relative to the time it takes until mutant cells take over the population. Hence, we assume in the following that $(x_1, \dots, x_{n-1})^T$ is proportional to the eigenvector of $(m_{i\to j} - \delta_{ji})_{ji\in\{1,\dots,n-1\}}$ corresponding to the dominant eigenvalue ξ, which depends on the copy number n and on the mode of replication (see Eq. (A.13) and (A.18), $m_{i\to j}$ defined in (A.12) denotes the expected number of j-type cells produced at division of a i-type cell). Once equilibrated, the frequencies of the heterozygous cells relative to each other χ_j remain constant throughout

the entire fixation process, which can formally be seen by the following calculation:

$$
\begin{aligned}
\frac{\mathrm{d}\frac{x_j}{x_{\text{het}}}}{\mathrm{d}t} &= \frac{\dot{x}_j}{x_{\text{het}}} - \frac{x_j}{x_{\text{het}}^2}\dot{x}_{\text{het}} \\
&= \frac{1}{x_{\text{het}}}\left\{\dot{x}_j - \frac{x_j}{x_{\text{het}}}x_{\text{het}}\right\} \\
&= \frac{1}{x_{\text{het}}}\left\{-x_0x_j - (1+s)x_nx_j + \sum_{i=1}^{n-1} x_i(1+s)(m_{i\to j} - x_j) - x_j(1+s) - \frac{x_j}{x_{\text{het}}}\sum_{k=1}^{n-1}\dot{x}_k\right\} \\
&= \frac{1}{x_{\text{het}}}\Bigg\{ - x_0x_j - (1+s)x_nx_j + \sum_{i=1}^{n-1} x_i(1+s)m_{i\to j} - (1+s)x_jx_{\text{het}} - x_j(1+s) \\
&\qquad - \frac{x_j}{x_{\text{het}}}\sum_{k=1}^{n-1}\left(-x_0x_k - (1+s)x_nx_k\sum_{i=1}^{n-1}x_i(1+s)(m_{i\to k} - x_k) - x_k(1+s)\right)\Bigg\} \\
&= \frac{1}{x_{\text{het}}}\Bigg\{ - x_0x_j - (1+s)x_j(1-x_0) + \sum_{i=1}^{n-1}(1+s)x_i(m_{i\to j} - \delta_{ij}) \\
&\qquad - \frac{x_j}{x_{\text{het}}}\sum_{k=1}^{n-1}\left(-x_0x_k - (1+s)x_k(1-x_0) + \sum_{i=1}^{n-1}x_i(1+s)(m_{i\to k} - \delta_{ik})\right)\Bigg\} \\
&= \frac{1}{x_{\text{het}}}\Bigg\{ - x_0x_j - (1+s)x_j(1-x_0) + \sum_{i=1}^{n-1}(1+s)x_i(m_{i\to j} - \delta_{ij}) \\
&\qquad - \frac{x_j}{x_{\text{het}}}\left(-x_0x_{\text{het}} - (1+s)x_{\text{het}}(1-x_0) + \sum_{k=1}^{n-1}\sum_{i=1}^{n-1}x_i(1+s)(m_{i\to k} - \delta_{ik})\right)\Bigg\} \\
&= \frac{1}{x_{\text{het}}}\left\{\sum_{i=1}^{n-1}(1+s)x_i(m_{i\to j} - \delta_{ij}) - \frac{x_j}{x_{\text{het}}}\sum_{k=1}^{n-1}\sum_{i=1}^{n-1}x_i(1+s)(m_{i\to k} - \delta_{ik})\right\} \\
&= (1+s)\left\{\sum_{i=1}^{n-1}\frac{x_i}{x_{\text{het}}}(m_{i\to j} - \delta_{ij}) - \frac{x_j}{x_{\text{het}}}\sum_{k=1}^{n-1}\sum_{i=1}^{n-1}\frac{x_i}{x_{\text{het}}}(m_{i\to k} - \delta_{ik})\right\} \\
&= (1+s)\left\{\xi\frac{x_j}{x_{\text{het}}} - \frac{x_j}{x_{\text{het}}}\sum_{k=1}^{n-1}\xi\frac{x_k}{x_{\text{het}}}\right\} \\
&= (1+s)\left\{\xi\frac{x_j}{x_{\text{het}}} - \frac{x_j}{x_{\text{het}}}\xi\right\} \\
&= 0. \qquad\qquad \text{(S1.1)}
\end{aligned}
$$

The evolution of the population through time in our model can then be described by a

system of three ordinary differential equations for the frequency of the wild type x_0, the sum of frequencies of all heterozygous types $x_{\text{het}} = x_1 + \cdots + x_{n-1}$, and frequency of the homozygous mutant type x_n. From equation (A.11), we obtain for the time-derivative of the homozygous mutant type $i = n$

$$
\begin{aligned}
\dot{x}_n &= \sum_{i=0}^{n} \{x_i \lambda_i (m_{i\to n} - x_n)\} - x_i \lambda_n \\
&= x_0(m_{0\to n} - x_n) + \sum_{i=1}^{n-1} x_i(1+s)(m_{i\to n} - x_n) + x_n(1+s)(m_{n\to n} - x_n) - x_n(1+s) \\
&= -x_0 x_n + (1+s)\left(\sum_{i=1}^{n-1} m_{i\to n}\chi_i x_{\text{het}} - x_n \sum_{i=1}^{n-1} x_i\right) + x_n(1+s)(2 - x_n) - x_n(1+s) \\
&= -x_0 x_n + (1+s)\kappa x_{\text{het}} - (1+s)x_{\text{het}} x_n - (1+s)x_n^2 + (1+s)x_n \\
&= -(1 - x_{\text{het}} - x_n) + (1+s)\kappa x_{\text{het}} - (1+s)x_{\text{het}} x_n - (1+s)x_n^2 + (1+s)x_n \\
&= -s x_n^2 - s x_{\text{het}} x_n + (1+s)\kappa x_{\text{het}} + s x_n,
\end{aligned}
$$

where we used $m_{0\to n} = 0$ and $m_{n\to n} = 2$, and defined $\kappa \coloneqq \sum_{i=1}^{n-1} m_{i\to n} \frac{x_i}{x_{\text{het}}}$. It holds

$$
\begin{aligned}
\kappa &= \sum_{i=1}^{n-1} (m_{i\to n} - \delta_{in})\chi_i \\
&= \sum_{j=0}^{n} \sum_{i=1}^{n-1} (m_{i\to j} - \delta_{ij})\chi_i - \sum_{j=0}^{n-1} \sum_{i=1}^{n-1} (m_{i\to j} - \delta_{ij})\chi_i \\
&= 1 - \sum_{i=1}^{n-1} (m_{i\to 0} - \delta_{ij})\chi_i - \sum_{j=1}^{n-1} \sum_{i=1}^{n-1} (m_{i\to j} - \delta_{ij})\chi_i \\
&= 1 - \sum_{i=1}^{n-1} (m_{i\to n} - \delta_{ij})\chi_i - \xi \\
&= 1 - \kappa - \xi,
\end{aligned}
$$

where we have used $\sum_{i=1}^{n-1} \sum_{j=0}^{n} (m_{i\to j} - \delta_{ij})\, \chi_i = 1$ (every cell has two daughter cells) and

the symmetry $m_{i\to j} = m_{n-i\to n-j}$. We therefore obtain

$$\kappa = \frac{1}{2}(1-\xi). \tag{S1.2}$$

For the sum of heterozygous cells, we obtain

$$\begin{aligned}
\dot{x}_{\text{het}} &= \sum_{i=1}^{n-1} \dot{x}_i \\
&= \sum_{i=1}^{n-1} \left(\sum_{k=0}^{n} x_k \lambda_k (m_{k\to i} - x_i) - x_i \lambda_i \right) \\
&= \sum_{i=1}^{n-1} \left(x_0 (m_{0\to i} x_i) + \sum_{k=1}^{n-1} x_k (1+s)(m_{k\to i} - x_i) + x_n (1+s)(m_{n\to i} - x_i) - x_i(1+s) \right) \\
&= \sum_{i=1}^{n-1} \left(-x_0 x_i + (1+s) \left(\sum_{k=1}^{n-1} m_{k\to i} \chi_k x_{\text{het}} - x_i \sum_{k=1}^{n-1} x_k \right) - x_n (1+s) x_i - x_i (1+s) \right) \\
&= \sum_{i=1}^{n-1} \left(-x_0 x_{\text{het}} + (1+s)(\xi+1)\chi_i x_{\text{het}} - (1+s) x_i x_{\text{het}} - x_n (1+s) x_i - x_i (1+s) \right) \\
&= -x_0 x_{\text{het}} + (1+s)(\xi+1) x_{\text{het}} - (1+s) x_{\text{het}}^2 - x_{\text{het}} (1+s) x_n - x_{\text{het}} (1+s) \\
&= -(1 - x_{\text{het}} - x_n) x_{\text{het}} + (1+s)(\xi+1) x_{\text{het}} + (1+s) x_{\text{het}}^2 - x_n (1+s) x_{\text{het}} - x_{\text{het}} (1+s) \\
&= -s x_{\text{het}}^2 - s x_n x_{\text{het}} + \left((1+s)\xi - 1 \right) x_{\text{het}}.
\end{aligned}$$

To make progress, it is easier to switch variables and to consider the total frequency of mutant cells $x_{\text{mut}} \coloneqq x_{\text{het}} + x_{\text{n}}$ and the relative fraction of heterozygous cells among all

mutant cells $x_{\mathrm{hf}} \coloneqq \frac{x_{\mathrm{het}}}{x_{\mathrm{mut}}}$ instead of x_{het} and x_n. The time-derivative of x_{mut} is given by

$$
\begin{aligned}
\frac{\mathrm{d}x_{\mathrm{mut}}}{\mathrm{d}t} &= \dot{x}_{\mathrm{het}} + \dot{x}_n \\
&= -sx_{\mathrm{het}}^2 - sx_n^2 - 2sx_nx_{\mathrm{het}} + \left((1+s)(\xi+\kappa)-1\right)x_{\mathrm{het}} + sx_n \\
&= -s(x_{\mathrm{het}}+x_n)^2 + \left((1+s)(\xi+\kappa)-1\right)x_{\mathrm{het}} \\
&= -sx_{\mathrm{mut}}^2 + \left((1+s)(\xi+\kappa)-1\right)x_{\mathrm{mut}}x_{\mathrm{hf}} + sx_{\mathrm{mut}}(1-x_{\mathrm{hf}}) \qquad \text{(S1.3a)} \\
&= -sx_{\mathrm{mut}}^2 + sx_{\mathrm{mut}} + \underbrace{(1+s)(\xi+\kappa-1)}_{=:a}x_{\mathrm{mut}}x_{\mathrm{hf}}. \qquad \text{(S1.3b)}
\end{aligned}
$$

For the time-derivative of x_{hf}, we obtain

$$
\begin{aligned}
\dot{x}_{\mathrm{hf}} &= \frac{\mathrm{d}}{\mathrm{d}t}\left(\frac{x_{\mathrm{het}}}{x_{\mathrm{mut}}}\right) \\
&= \frac{\dot{x}_{\mathrm{het}}}{x_{\mathrm{mut}}} - \frac{x_{\mathrm{hf}}}{x_{\mathrm{mut}}}\dot{x}_{\mathrm{mut}} \\
&= \frac{-sx_{\mathrm{het}}^2 - sx_nx_{\mathrm{het}} + \left((1+s)\xi-1\right)x_{\mathrm{het}}}{x_{\mathrm{mut}}} \\
&\quad - \frac{x_{\mathrm{hf}}}{x_{\mathrm{mut}}}\left(-sx_{\mathrm{mut}}^2 + \left((1+s)(\xi+\kappa)-1\right)x_{\mathrm{mut}}x_{\mathrm{hf}} + sx_{\mathrm{mut}}(1-x_{\mathrm{hf}})\right) \\
&= -sx_{\mathrm{het}}x_{\mathrm{hf}} - sx_nx_{\mathrm{hf}} + \left((1+s)\xi-1\right)x_{\mathrm{hf}} \\
&\quad + sx_{\mathrm{hf}}x_{\mathrm{mut}} - \left((1+s)(\xi+\kappa)-1\right)x_{\mathrm{hf}}^2 - s(1-x_{\mathrm{hf}})x_{\mathrm{hf}} \\
&= -sx_{\mathrm{mut}}x_{\mathrm{hf}} + \left((1+s)\xi-1\right)x_{\mathrm{hf}} \\
&\quad + sx_{\mathrm{hf}}x_{\mathrm{mut}} - \left((1+s)(\xi+\kappa)-1\right)x_{\mathrm{hf}}^2 - s(1-x_{\mathrm{hf}})x_{\mathrm{hf}} \\
&= ((1+s)\xi-1-s)x_{\mathrm{hf}} + (s+1-(1+s)(\xi+\kappa))\,x_{\mathrm{hf}}^2 \\
&= -\underbrace{(1+s)(\xi+\kappa-1)}_{=a}x_{\mathrm{hf}}^2 + \underbrace{(1+s)(\xi-1)}_{=:b}x_{\mathrm{hf}},
\end{aligned}
$$

where we have used Eq. (S1.3a). The general solution to this Bernoulli differential equation

(Zeidler, 2013) is

$$x_{\mathrm{hf}}(t) = \frac{be^{bC+bt}}{ae^{bC+bt}+1}. \tag{S1.4}$$

From the initial condition $x_1 = f, x_2 = 0, \ldots, x_n = 0$, such that $x_{\mathrm{hf}} = \frac{x_{\mathrm{het}}}{x_{\mathrm{mut}}} = 1$, we get $C = \frac{\ln\left(\frac{1}{b-a}\right)}{b}$. Substituting C into Eq. (S1.4) yields

$$\begin{aligned} x_{\mathrm{hf}}(t) &= \frac{b(\frac{1}{b-a})e^{bt}}{a(\frac{1}{b-a})e^{bt}+1} \\ &= \frac{be^{bt}}{ae^{bt}+b-a} \qquad \text{(S1.5a)} \\ &= \frac{(1+s)(\xi-1)e^{(1+s)(\xi-1)t}}{(1+s)(1-\xi-\kappa)e^{(1+s)(\xi-1)t}-(1+s)\kappa} \\ &= \frac{(\xi-1)e^{(1+s)(\xi-1)t}}{(1-\xi-\kappa)e^{(1+s)(\xi-1)t}-\kappa}. \qquad \text{(S1.5b)} \end{aligned}$$

Inserting Eq. (S1.5a) into (S1.3b) gives

$$\dot{x}_{\mathrm{mut}} = -sx_{\mathrm{mut}}^2 + sx_{\mathrm{mut}} + ax_{\mathrm{mut}}\frac{be^{bt}}{ae^{bt}+b-a}. \tag{S1.6}$$

For the initial condition $x_{\mathrm{mut}}(0) = f$, the solution (obtained with Mathematica Version 12.0.0.0 (Wolfram Research, Inc.)) is given by

$$\begin{aligned} x_{\mathrm{mut}}(t) &= \frac{f(b+s)e^{st}\left(a\left(e^{bt}-1\right)+b\right)}{fe^{st}\left(ase^{bt}-(a-b)(b+s)\right)+b(af+b(-f)+b-fs+s)} \\ &= \frac{f(\xi+\xi s-1)e^{st}\left(\kappa-(\kappa+\xi-1)e^{(\xi-1)(s+1)t}\right)}{\begin{array}{l} f\kappa(\xi-1)\left(e^{st}-1\right) \\ \quad + fs\left(\kappa+\xi+\kappa\xi\left(e^{st}-1\right)-(\kappa+\xi-1)e^{t(\xi+\xi s-1)}-1\right)-(\xi-1)(\xi+\xi s-1) \end{array}}. \end{aligned} \tag{S1.7}$$

In the next step, we derive an expression for the size of the heterozygosity window in the limit $x_{\mathrm{thr}} \to 1$, where x_{thr} denotes the threshold for fixation, and show that it is independent

of the initial frequency f. The limit $x_{\mathrm{thr}} \to 1$ implies that t_{fix} and t_{phen} both tend to infinity, and we thus study the behavior of the system for large times (formally $t \to \infty$).

For the time to fixation at the phenotype level t_{phen}, it holds that $x_0(t_{\mathrm{phen}}) = 1 - x_{\mathrm{mut}}(t_{\mathrm{phen}}) = 1 - x_{\mathrm{thr}}$. For the time to fixation at the genotype level t_{fix} (fixation of homozygous mutant cells), it analogously holds $x_0(t_{\mathrm{fix}}) + x_{\mathrm{het}}(t_{\mathrm{fix}}) = 1 - x_{\mathrm{n}}(t_{\mathrm{fix}}) = 1 - x_{\mathrm{thr}}$, where we have $x_{\mathrm{wt}} \coloneqq x_0 + x_{\mathrm{het}}$ as the frequency of cells that carry wild-type replicon copies. Combining the latter equations, we get

$$1 - x_{\mathrm{thr}} = x_0(t_{\mathrm{phen}}) = x_{\mathrm{wt}}(t_{\mathrm{fix}}). \tag{S1.8}$$

If the strength of selection is large compared to the inverse copy number, so that we expect a heterozygosity window (cf. the threshold of Eq. (1)), the decay rate of the frequency of wild-type carrying cells $x_{\mathrm{wt}}(t)$ is approximately given by

$$\frac{1}{t}\ln x_{\mathrm{wt}}(t) = \frac{1}{t}\ln\left(1 - x_{\mathrm{n}}(t)\right) = \frac{1}{t}\ln\left(1 - x_{\mathrm{mut}}(t)(1 - x_{\mathrm{hf}}(t))\right) \overset{t\to\infty}{\longrightarrow} (1+s)(\xi - 1), \tag{S1.9}$$

where we obtained the limit using MATHEMATICA as above (see File S2). MATHEMATICA states the condition $\xi > \frac{2+s}{2(1+s)}$ as a condition for this limit, which is is equivalent to $s > \frac{2}{n-5/2} \approx \frac{2}{n}$ for regular replication and $s > (8n)/(-1 - 7n + 2n^2) \approx \frac{4}{n}$ for random replication. This is more stringent than the condition for the existence of a heterozygosity window (Eq. (1)), which is $s \gtrsim \frac{1}{n}$ (regular replication, Eq. (1)) or $s \gtrsim \frac{2}{n}$ (random replication, Eq. (2)). Numerical results for relevant cases of n and s, however, show that Eq. (S1.9) also holds true if $\frac{1}{n} \lesssim s \lesssim \frac{2}{n}$ (see File S2). Thus, for large times, we have

$$x_{\mathrm{wt}}(t) \propto e^{(1+s)(\xi-1)t}.$$

Consequently, we have

$$x_{\mathrm{wt}}(t_{\mathrm{fix}}) = x_{\mathrm{wt}}(t_{\mathrm{phen}})e^{(1+s)(\xi-1)\Delta t}, \tag{S1.10}$$

where we used the definition of the heterozygosity window $\Delta t = t_{\mathrm{fix}} - t_{\mathrm{phen}}$. Moreover, using again MATHEMATICA, we obtain for the limit

$$\frac{x_{\mathrm{wt}}(t)}{x_0(t)} \xrightarrow{t\to\infty} \frac{\kappa + (\kappa-1)s}{(1+s)(\kappa+\xi-1)} = \frac{2s}{(1+s)(1-\xi)} - 1 =: r, \tag{S1.11}$$

which is independent of f. Inserting Eq. (S1.10) and (S1.11) into Eq. (S1.8) gives, for the limit of the fixation threshold $x_{\mathrm{thr}} \to 1$,

$$x_{\mathrm{wt}}(t_{\mathrm{phen}})e^{(1+s)(\xi-1)\Delta t} = x_{\mathrm{wt}}(t_{\mathrm{fix}}) = x_0(t_{\mathrm{phen}}) = \frac{x_{\mathrm{wt}}(t_{\mathrm{phen}})}{r} \tag{S1.12}$$

$$\Leftrightarrow \qquad e^{(1+s)(\xi-1)\Delta t} = \frac{1}{r}$$

$$\Leftrightarrow \qquad \Delta t = \frac{\ln\frac{1}{r}}{(1+s)(\xi-1)} = \frac{\ln\left(\frac{(1+s)(\kappa+\xi-1)}{\kappa+(\kappa-1)s}\right)}{(1+s)(\xi-1)}, \tag{S1.13}$$

which is independent of the initial frequency f. Fig. S1 and S2 show comparisons to numerical simulations.

From (S1.12), we obtain $x_{\mathrm{wt}}(t_{\mathrm{phen}}) \approx r x_0(t_{\mathrm{phen}}) = r x_{\mathrm{thr}}$ for x_{thr} close to one. Thus, the frequency of heterozygotes at the time of phenotypic fixation can be approximated for large x_{thr} by

$$x_{\mathrm{het}}(t_{\mathrm{phen}}) = (r-1)x_{\mathrm{thr}}. \tag{S1.14}$$

Note that the condition $(1+s)\xi > 1$ (cf. Eq. (A.15)), which is required in the derivation, is equivalent to $r > 1$. A comparison to numerical simulations is shown in Fig. S2.

For the mode of regular replication, we obtain for the heterozygosity window by insertion

of the corresponding expressions for ξ and κ (Eqs. (A.13) and (S1.2))

$$\Delta t = \frac{2n-1}{2(1+s)} \ln\left(\frac{2(n-1)s-1}{s+1}\right) \tag{S1.15a}$$

$$\approx \frac{n}{1+s} \ln\left(\frac{2ns}{1+s}\right). \tag{S1.15b}$$

Under the mode of random replication, we obtain analogously using Eqs. (A.18) and (S1.2)

$$\Delta t = \frac{2n^2+n-1}{4n(1+s)} \ln\left(\frac{n(2ns-s-2)-s}{2n(1+s)}\right) \tag{S1.16a}$$

$$\approx \frac{n/2}{1+s} \ln\left(\frac{ns}{1+s}\right). \tag{S1.16b}$$

Supplementary figures

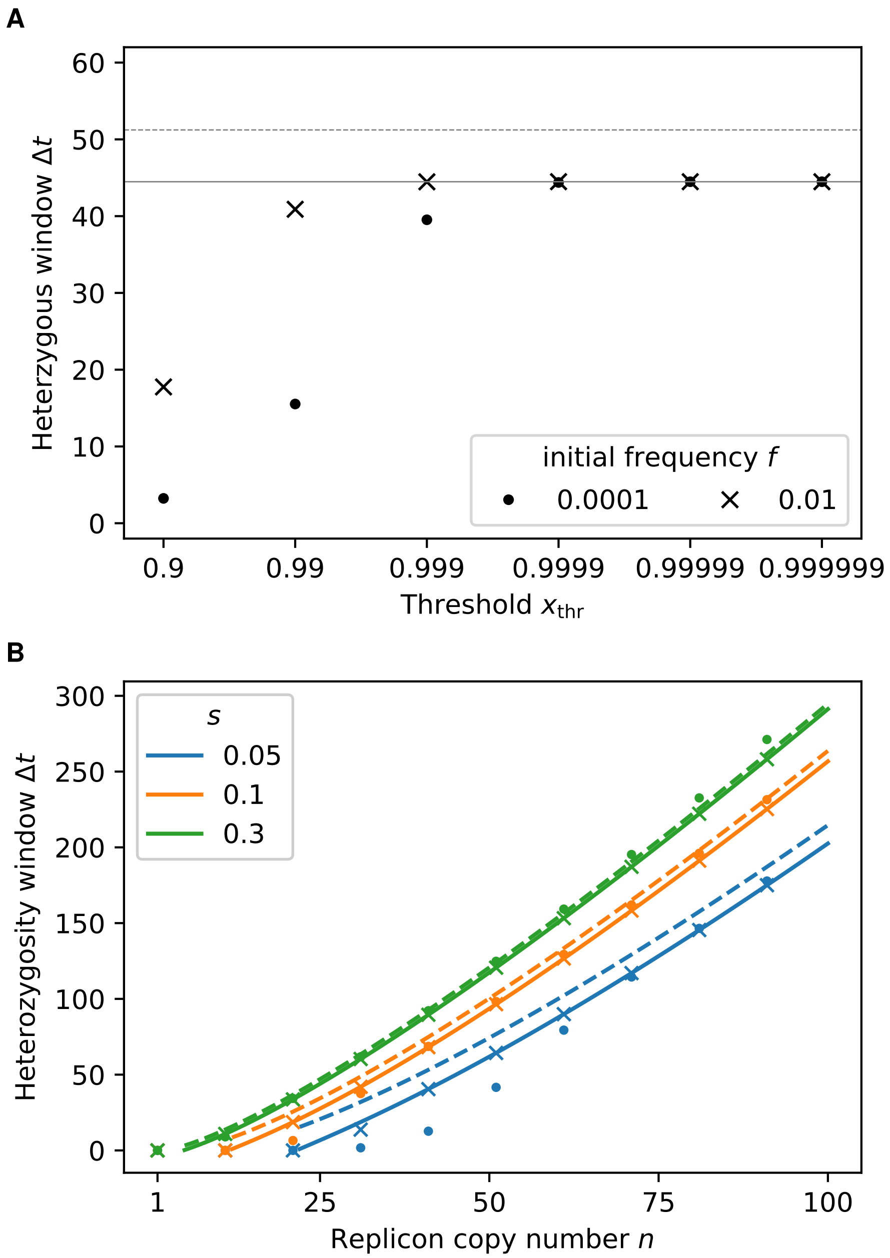

Figure S1: (A) Influence of the fixation threshold x_{thr} on the length of the heterozygosity window Δt. In the main text, a frequency of 99% mutant cells and of 99% homozygous mutant cells is the proxy for determining the fixation times t_{phen} and t_{fix} respectively. The plot shows the heterozygosity window $\Delta t = t_{\text{fix}} - t_{\text{phen}}$ obtained from the deterministic model (Eq. (A.11)) for various thresholds x_{thr} (markers) and from the analytical approximations (solid and dashed lines, showing Eqs. (S1.15a) and (S1.15b), respectively). For smaller initial frequencies of mutant cells f (see legend), the heterozygosity window converges later, i.e., for thresholds x_{thr} closer to 1. Parameters: replicon copy number $n = 32$, strength of selection $s = 0.1$, mode of regular replication. (B) Comparison of the length of the heterozygous window Δt for the threshold $x_{\text{thr}} = 99\%$ (dots) and $x_{\text{thr}} = 99.9999\%$ (crosses) with the analytical approximations (solid and dashed lines, showing Eqs. (S1.15a) and (S1.15b), respectively) for various replicon copy numbers n and strength of selection s. Parameters: initial frequency of mutant cells $f = 1\%$, mode of regular replication.

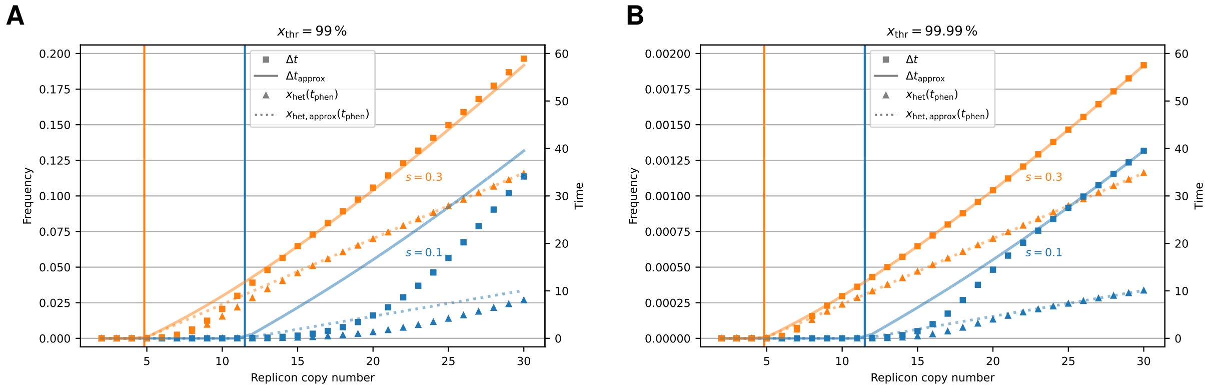

Figure S2: Influence of the replicon copy number n and the strength of selection s on the remaining frequency of heterozygotes at the phenotypic fixation time, $x_{\mathrm{het}}(t_{\mathrm{phen}})$, and the heterozygosity window Δt, assuming regular replication and random segregation. Thin lines show results from the numerical integration of Eq. (A.11), and thick lines show the analytical approximations of $x_{\mathrm{het}}(t_{\mathrm{phen}})$ (Eq. (S1.14)) and Δt (Eq. (S1.15a)) for small initial frequencies f and $x_{\mathrm{thr}} \approx 1$. (A) and (B) show results for two different fixation thresholds (99 % and 99.99 %).

A

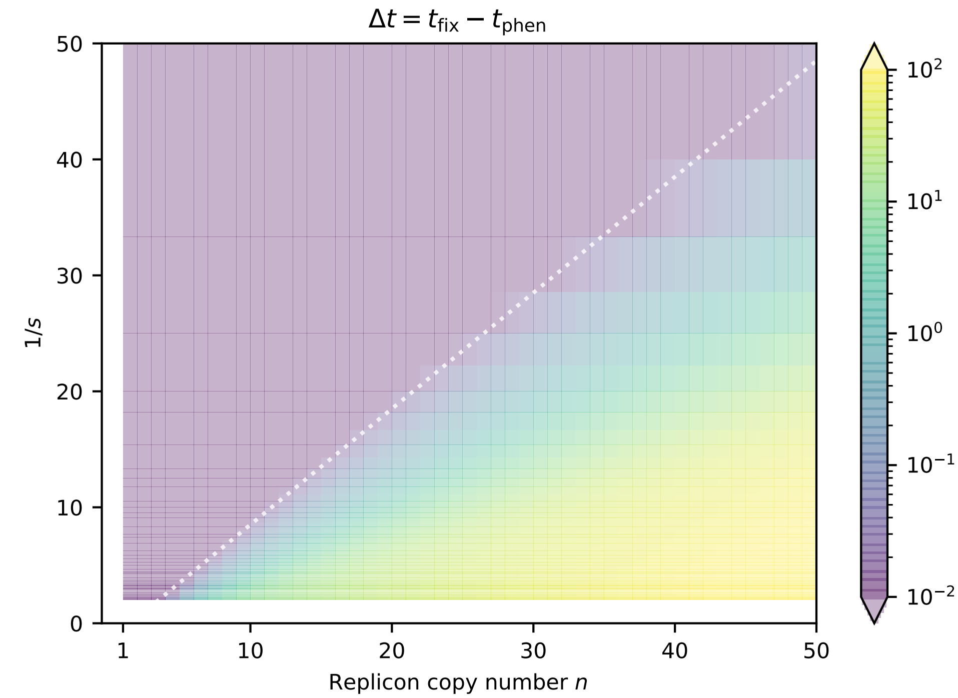

B

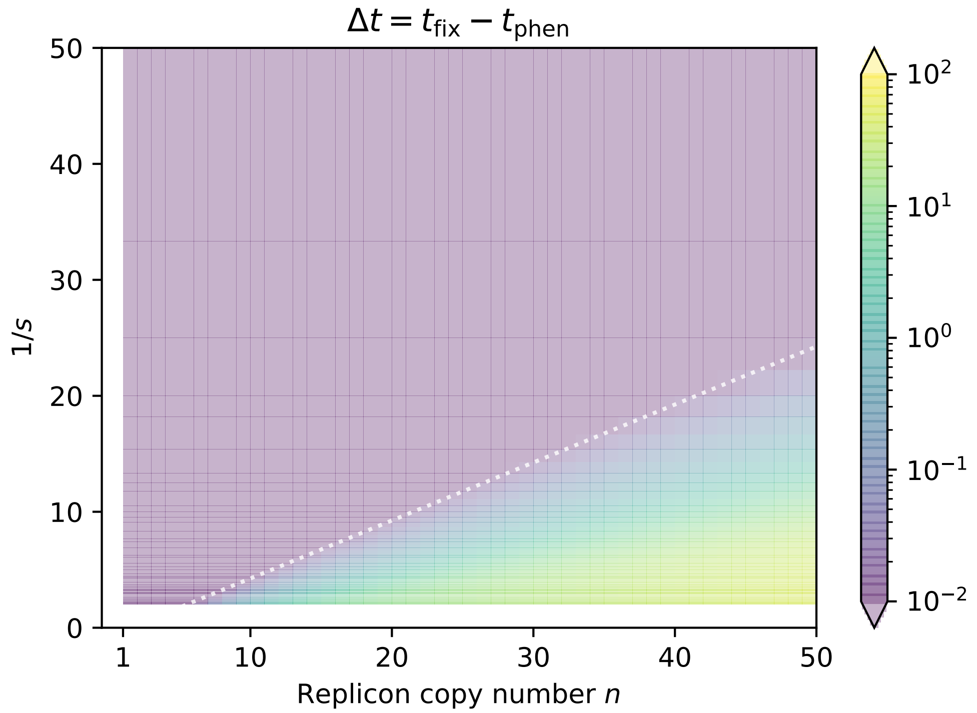

Figure S3: The heterozygosity window Δt for various combinations of the replicon copy number n and the inverse of the selective advantage $1/s$ for the mode of random segregation with (A) regular replication and (B) random replication. The initial frequency of mutant cells with one mutant replicon copy is $f = 0.01$. The dotted lines denote the threshold above which a heterozygosity windows arises given by Eq. (1) and (2) respectively.

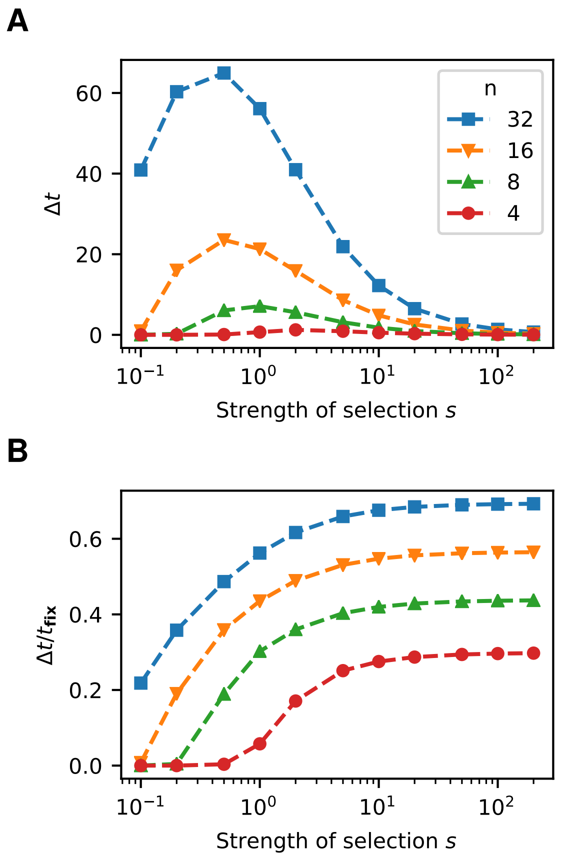

Figure S4: The heterozygosity window as a function of the strength of selection s (x-axis) for various replicon copy numbers n (lines). Panel A shows the absolute size Δt and Panel B the size relative to the phenotypic fixation time $\Delta t/t_{\mathrm{phen}}$. Both the absolute and the relative sizes increase with n. The relative size of the heterozygosity window (Panel B) also monotonically increases with the strength of selection s. The absolute size (Panel A) has a maximum as a function of s, since the fixation times become shorter with increasing s, which ulimately also affects the size of the window.

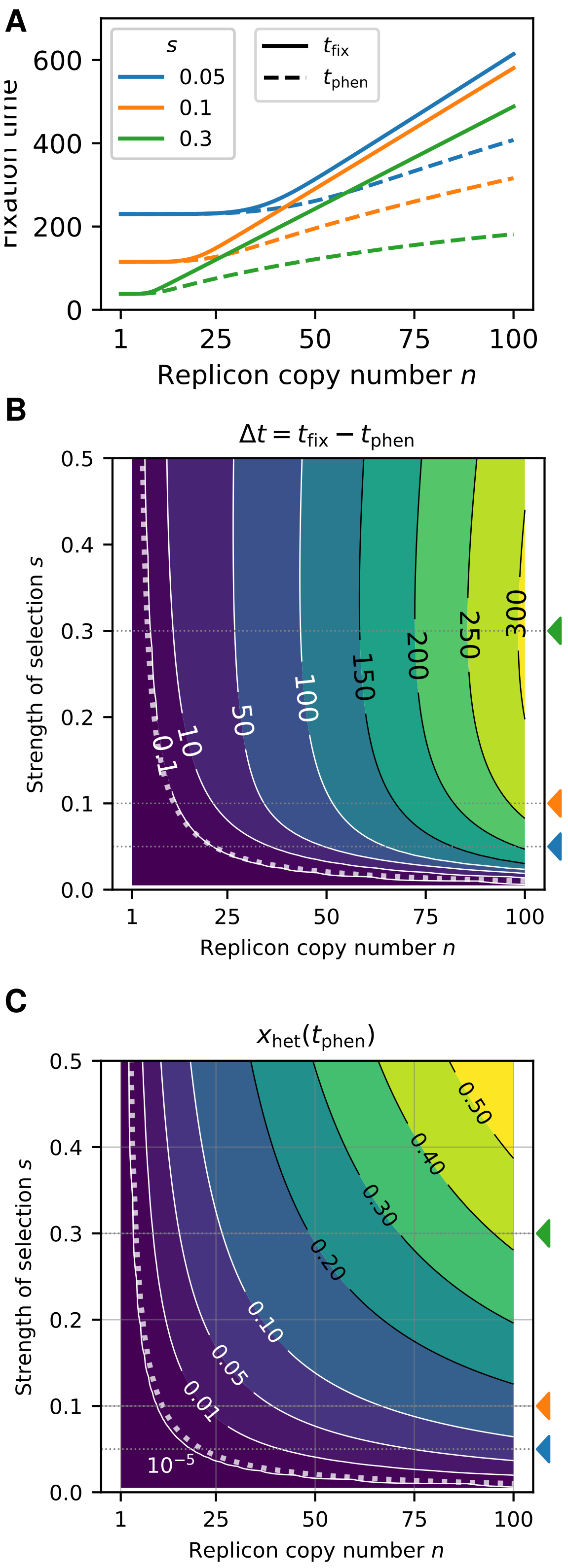

Figure S5: Influence of the replicon copy number n and the strength of selection s on the fixation times and the heterozygosity window for constant initial frequencies of mutant replicon copies $f_{\text{rep}} = f/n = 0.001$. The initial frequency of mutant cells with one mutant replicon copy is set to $f = f_{\text{rep}}n$. The panels are analogous to those of Figure 3, where f rather than f_{rep} is kept constant. (A) Fixation times as a function of the replicon copy number for several selection coefficients $s = 0.05$ (blue), 0.1 (orange), 0.3 (green) (B) Contour plot of the heterozygosity window for various replicon copy numbers n and selection coefficients s. (C) Frequency of remaining heterozygotes at the time point of phenotypic fixation t_{phen}.

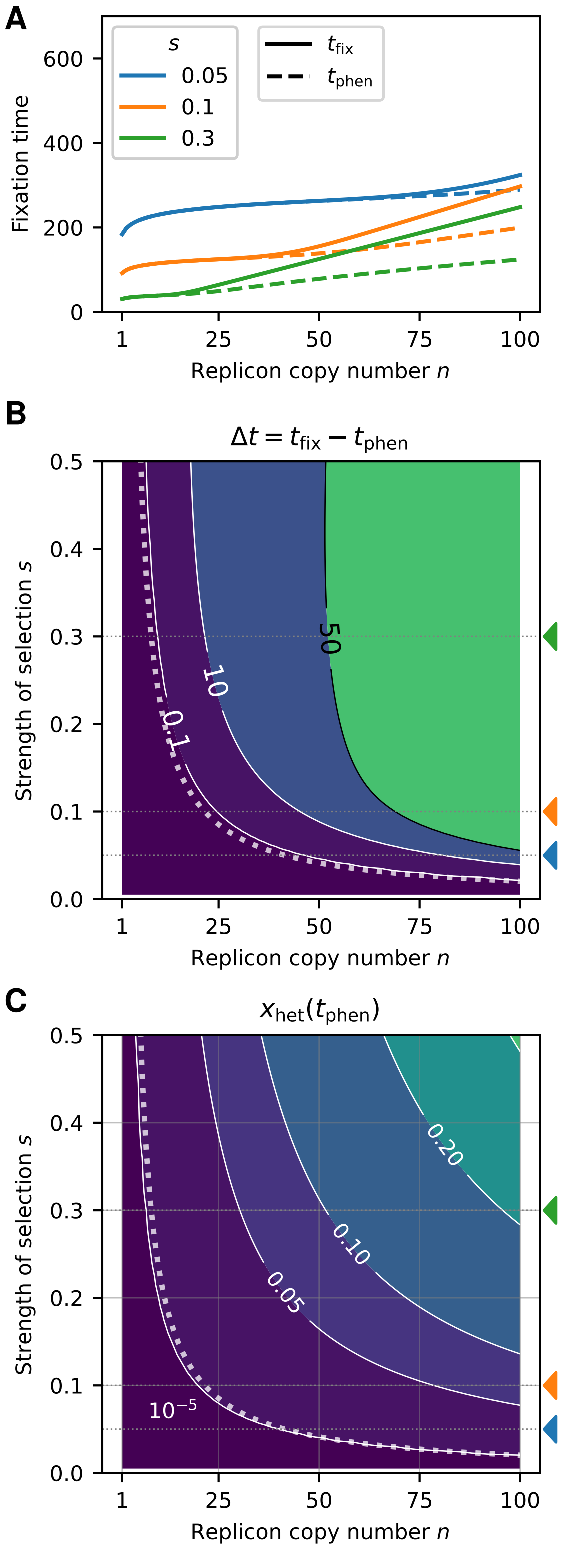

Figure S6: Influence of the replicon copy number n and the strength of selection s on the fixation times and the heterozygosity window for a replicon subject to random replication and random segregation. This figure is analogous to Figure 3, considering random replication instead of regular replication of replicon copies. The dotted line in (C) shows the threshold for s at which the heterozygosity window start to occur (criterion (2)).

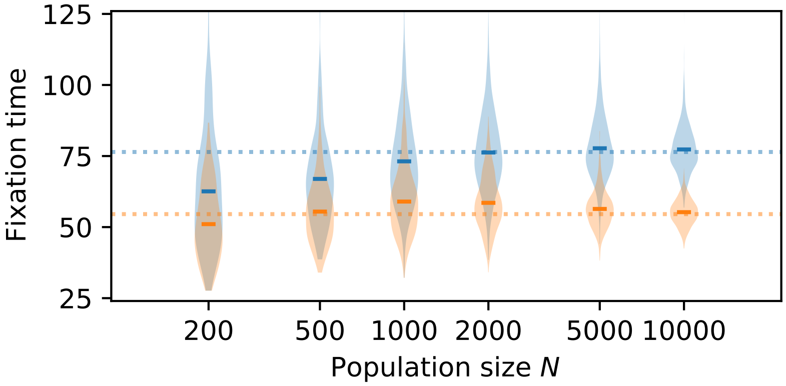

Figure S7: Fixation times of mutant cells t_{phen} (orange) and of homozygous mutant cells t_{fix} (blue) for various population sizes N. Violin plots show the distribution from 10^3 stochastic simulations. Horizontal lines within the violin plots indicate the mean fixation times. The dashed horizontal lines show results from the deterministic model (Eq. (A.11)) reflecting an infinite population. Parameters: $n = 16$, $s = 0.3$, $f = 0.01$.

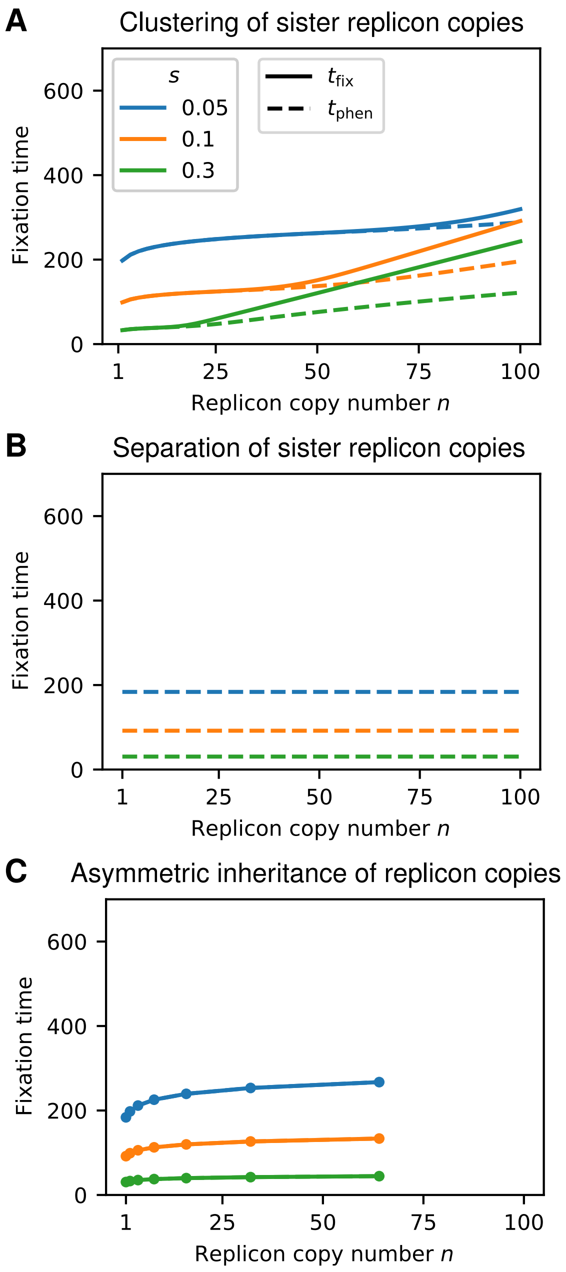

Figure S8: Fixation times as a function of the replicon copy number of simulations for several selection coefficients $s = 0.05$ (blue), 0.1 (orange), 0.3 (green) using the alternative segregation modes. The plots are analogous to Figure 3A (baseline model with *random segregation*) and show results for (A) *clustering of sister replicon copies*, (B) *separation of sister replicon copies*, and (C) *asymmetric inheritance of replicon copies*. Parameter: $f = 0.01$.

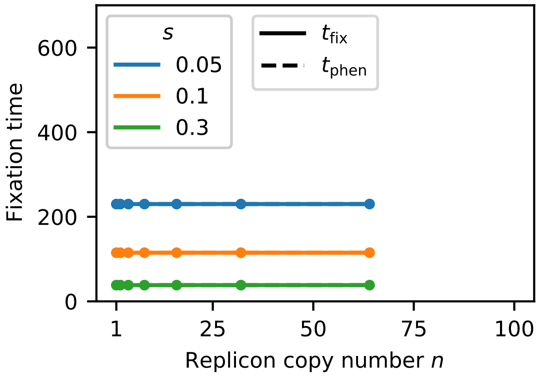

Figure S9: Influence of the replicon copy number n and the strength of selection s on the fixation times for constant initial frequencies of mutant replicon copies $f_{\mathrm{rep}} = f/n = 0.001$ for *asymmetric inheritance of replicon copies*. The figure is analogous to Figure S5A.

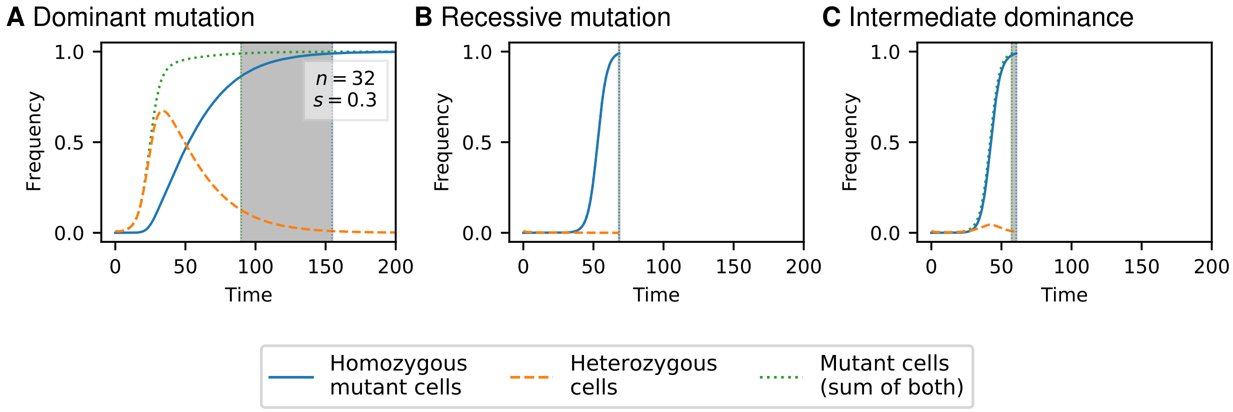

Figure S10: Frequency trajectories of different cell types for mutations of various dominance, assuming random segregation and regular replication. The figure is analogous to Figure 2D. (A) Cells carrying at least one mutant replicon copy have a selective advantage s (as in the main text, same plot as in Figure 2). (B) Cells carrying only mutant replicon copies have a selective advantage s. (C) The selective advantage of cells carrying i mutant copies is given by $s\frac{i}{n}$. Parameters: Replicon copy number $n = 32$, strength of selection $s = 0.3$. The time unit corresponds to the generation time of the wild type.

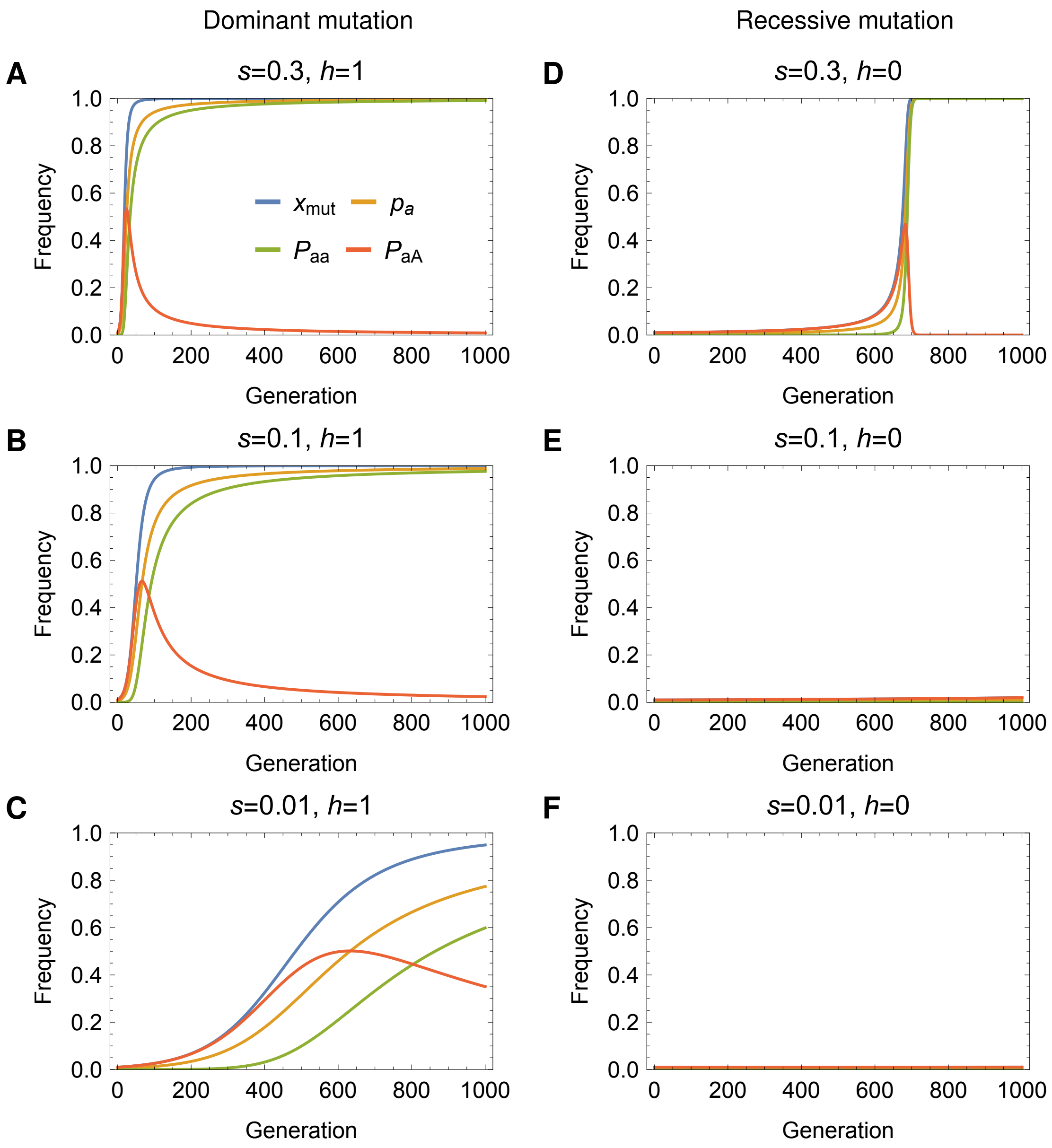

Figure S11: The spread of a beneficial dominant allele (Panels A-D, $h = 1$) and of a beneficial recessive allele (Panels E-H, $h = 0$) in a sexually reproducing population with random mating. The dynamics are modeled by a deterministic Wright-Fisher model with selection (Etheridge, 2011). The variable p_a denotes the frequency of the beneficial allele a in the population. Genotype frequencies of homozygous mutant and heterozygous cells in the subsequent generation are given by $P_{aa} = \frac{p_a^2(1+s)}{\bar{w}}$ and $P_{aA} = \frac{2p_a(1-p_a)(1+hs)}{\bar{w}}$ respectively, where $\bar{w} = 1 + sp_a^2 + 2hsp_a(1-p_a)$. Phenotypically mutant cells have a fraction $x_{\text{mut}} = P_{aa}+P_{aA}$ for dominant mutations and $x_{\text{mut}} = P_{aa}$ for recessive mutations in the population. The allele frequency is given by $p_a = P_{aa}+P_{aA}/2$. The mutant allele frequency at generation 0 is set to $p_a = f/2$ with $f = 0.01$.

References

Alison Etheridge. *Some Mathematical Models from Population Genetics*. Springer, 2011.

Wolfram Research, Inc. Mathematica, Version 12.0.0. URL https://www.wolfram.com/mathematica. Champaign, IL, 2021.

Eberhard Zeidler. *Oxford Users' Guide to Mathematics*. OUP Oxford, New York, London, 1 edition, 2013.
